# Personalized stress detection from wearable physiological signals via Digital Twin-inspired subject embeddings and recurrent neural networks

**DOI:** 10.3389/fphys.2026.1881803

**Published:** 2026-10-15

**Authors:** Hadeel Alharbi

**Affiliations:** Department of Artificial Intelligence and Data Science, College of Computer Science and Engineering, University of Ha’il, Hail, Saudi Arabia

**Keywords:** Digital Twin, GRU, multimodal fusion, personalization, stress detection, wearable sensing

## Abstract

**Introduction:**

The rising prevalence of psychological stress and its associated adverse health effects have created an urgent need for reliable and automated detection methods that move beyond traditional self-report tools. While wearable sensing systems offer a promising avenue for continuous monitoring, current systems often fail to account for individual physiological variability in stress responses. This leads to suboptimal performance when applied across diverse populations. To address this challenge, this paper presents a novel framework aimed at personalized stress detection in wearable sensing systems.

**Methods:**

We introduce a DT-GRU framework centered on a trainable subject-embedding module that captures each individual’s unique physiological traits, thereby forming a latent Digital Twin (DT). This personalized embedding is integrated with Gated Recurrent Unit (GRU) features in the model's inference pathway, enabling the framework to utilize both temporal dynamics and subject identity for improved detection. The DT-GRU framework was rigorously evaluated on the WESAD dataset, incorporating various validation techniques, including subject-independent train-test splits and Leave-One-Subject-Out (LOSO) cross-validation.

**Results:**

The results from the evaluation demonstrate significant improvements in classification accuracy and generalizability. The integration of the personalized subject-embedding was shown to enhance the model’s performance across the diverse population represented in the dataset.

**Discussion:**

These findings highlight the potential of the DT-GRU approach for deployment in real-world stress monitoring applications. By effectively accounting for individual physiological variability, the framework provides a more reliable and personalized method for automated stress detection, paving the way for more effective and scalable mental health monitoring solutions.

## 1 Introduction

Psychological stress is one of the most prevalent health challenges of the modern era, with well-documented consequences for cardiovascular, immunological, and cognitive functioning (Bolpagni et al., 2024; Lazarou and Exarchos, 2024). Traditional means of evaluating stress are primarily based on questionnaires, and one-off clinical evaluations largely depend on self-report tools, which are subjective, episodic, and cannot reflect the actual continuous dynamic physiological response to stressors in real life (Schmidt et al., 2018). The rapid development of inexpensive wrist-worn wearable devices has created an opportunity to continuously and objectively monitor stress using multimodal physiological signals, including electrodermal activity (EDA), blood volume pulse, skin temperature, and tri-axial acceleration, enabling unobtrusive and ecologically valid recognition of affective state (Moser et al., 2024). When stress becomes chronic and persists over time, it can lead to hypertension, depression, burnout, and more. Therefore, there is a pressing need for automated, reliable systems for detecting stress (Singh et al., 2024). Despite significant recent progress in wearable sensing and affective computing, the development of accurate, generalizable, and personalized stress detection frameworks remains an open research problem of considerable clinical significance.

There has been promising work on the automatic detection of stress from physiological time-series data using deep learning. Recurrent architectures like Long Short-Term Memory (LSTM) and Gated Recurrent Unit (GRU) networks have been applied to model temporal dependencies in physiological signals. Moreover, hybrid CNN-RNN models and attention-based frameworks have enhanced classification performance on benchmark datasets (Moser et al., 2024; Singh et al., 2024). The WESAD dataset, introduced by Schmidt et al. (2018) has emerged as a widely adopted standard for wearable stress and affect detection, providing synchronized multimodal physiological recordings from 15 subjects across three affective states under controlled laboratory conditions. Despite the progress enabled by such benchmarks, a fundamental limitation persists across existing approaches: models are typically trained in a subject-agnostic manner, learning population-level representations that fail to account for pronounced inter-subject physiological variability in stress responses (Moser et al., 2024). This variability means that the signal pattern indicative of stress in one individual may be entirely unremarkable in another, making personalization a critical yet largely unresolved requirement for clinically deployable wearable stress monitoring systems.

To address these limitations, this paper proposes a DT Enhanced Gated Recurrent Unit (DT-GRU) framework for personalized wearable stress detection. Inspired by the growing body of research establishing DT technology as a powerful paradigm for subject-specific physiological modeling in healthcare (Cellina et al., 2023; Vallée, 2023; Katsoulakis et al., 2024), we introduce a trainable subject embedding module that functions as a compact latent DT encoding the unique physiological characteristics of each individual directly into the model’s inference pathway. This embedding is concatenated with temporally encoded GRU features before classification, enabling the model to leverage both signal dynamics and subject identity simultaneously. The proposed framework is rigorously evaluated on the WESAD dataset using both subject-independent train-test splits and full Leave-One-Subject-Out (LOSO) cross-validation across all 15 subjects, providing a comprehensive assessment of within-cohort accuracy and cross-subject generalizability. The primary contributions of this work are summarized as follows:

- A DT-inspired subject-embedding module is proposed to encode individual physiological characteristics as a trainable latent representation, enabling subject-aware personalization within a unified end-to-end deep learning framework for wearable stress detection.
- A dual-branch architecture is designed that integrates a stacked GRU temporal encoder for modeling sequential physiological dynamics with a subject-specific DT embedding, fused via concatenation before binary stress classification.
- A rigorous two-stage evaluation protocol is established, combining subject-independent train-test splitting with full LOSO cross-validation across all 15 WESAD subjects, providing a transparent assessment of both accuracy and cross-subject generalizability.
- Subject-level personalization through DT embeddings is demonstrated to yield measurable improvements in classification performance over conventional subject-agnostic GRU baselines, confirming the utility of identity-conditioned modeling for wearable affective computing.
- Extensive experimental analysis and metric-level reporting, including AUC, F1-score, precision, and recall, alongside training curve visualizations and ROC analysis, establish a reproducible benchmark for DT-enhanced stress detection on the WESAD dataset.

The remainder of this paper is organized as follows. Section 2 reviews related work. Section 3 details the proposed methodology, including the dataset and preprocessing. Section 4 presents and discusses experimental results. Section 5 concludes the paper.

## 2 Related work

The automated detection of psychological stress from wearable physiological signals has attracted considerable research interest over the past decade, driven by the growing clinical imperative for continuous, objective, and unobtrusive mental health monitoring (Bolpagni et al., 2024). Electrodermal activity (EDA), blood volume pulse (BVP), skin temperature (TEMP), and accelerometry (ACC) have emerged as the most widely employed biosignals for this purpose, given their amenability to wrist-worn acquisition and their direct association with sympathetic nervous system arousal (Sosa et al., 2026). The use of biosensor-based stress monitoring systems has been further gaining traction with recent developments in wearable body area network (WBAN) systems. The combination of machine learning and optimization algorithms is becoming increasingly feasible for long-term wearable physiological monitoring systems in healthcare applications, as shown by Mohd Nazeer et al. (2024b), who demonstrated that machine-learning-based optimization strategies and unsupervised learning architectures can significantly enhance the longevity and network efficiency of biosensors in wireless body area networks (WBANs). The WESAD dataset, introduced by Schmidt et al. (2018), provided the first publicly available multimodal benchmark for wearable stress and affect detection, recording physiological data from 15 subjects across baseline, stress, and amusement conditions. This dataset established a reproducible evaluation standard against which subsequent models are commonly benchmarked and has been the foundational resource motivating the present study. Early approaches to stress classification on WESAD and related datasets predominantly employed handcrafted statistical and spectral features combined with classical machine learning classifiers such as linear discriminant analysis, support vector machines, and random forests, achieving moderate binary classification accuracies under LOSO evaluation (Schmidt et al., 2018). Mohd Nazeer et al (Mohd et al., 2023). evaluated the performance of classical machine learning methods in detecting stress using wearable biosensor data, concluding that systematic multimodal physiological feature engineering paired with supervised classifiers yields highly reliable stress recognition to obtain reliable stress recognition performance in real-time healthcare monitoring applications. While these methods established a useful baseline, their reliance on domain-expert feature engineering and their inability to capture complex temporal dynamics motivated the subsequent transition toward end-to-end deep learning frameworks. Recurrent neural network architectures, in particular Long Short-Term Memory (LSTM) and Gated Recurrent Unit (GRU) networks, have demonstrated strong performance in modeling sequential physiological signals, owing to their intrinsic capacity to capture temporal dependencies across variable-length time windows (Malviya et al., 2023). Singh et al. (2024) proposed a hybrid CNN-LSTM framework for the WESAD dataset within an IoMT context, achieving 90.45% binary classification accuracy by combining convolutional feature extraction with recurrent temporal modeling, outperforming prior machine learning and single-architecture deep learning approaches. Tanwar et al. (2024) introduced an attention-augmented CNN-LSTM model that dynamically prioritizes temporally salient features, demonstrating that attention mechanisms substantially enhance stress recognition by enabling selective focus on the most physiologically discriminative signal segments. Ensemble architectures further demonstrated the complementary strengths of LSTM and GRU for emotion and stress recognition in (Nandini et al., 2025), wherein stacked LSTM-GRU models consistently outperformed individual recurrent models across multimodal wearable physiological datasets. Modi et al. (2025) demonstrated that purely GRU-based temporal encoders, with appropriate dropout regularization, can match the performance of more complex hybrid architectures at a reduced computational cost, making them well-suited for resource-constrained wearable deployment.

Together, these works establish GRU networks as a computationally efficient and temporally expressive backbone for wearable stress detection, motivating their use as the temporal encoder in the present work. Gil-Martin et al. (2022) demonstrated that fusing EDA, BVP, TEMP, and ACC signals using convolutional architectures significantly improves detection accuracy over any individual modality, attributing the gains to reduced intermodal uncertainty. Yang et al. (2025) proposed transforming multimodal physiological signals, including EDA, ACC, BVP, and temperature, into Gramian Angular Field (GAF) and Markov Transition Field (MTF) image representations for CNN-based classification on WESAD, achieving an accuracy of 90.96% and an F1-score of 91.67%. Xiang et al. (2025) developed a parallel CNN architecture that processes time-domain and frequency-domain features extracted from EDA, ACC, HR, and TEMP signals simultaneously, demonstrating that incorporating spectral information substantially improves model robustness. Likewise, Nazeer et al. (2024a) investigated multimodal stress detection using a combination of electrocardiogram (ECG), galvanic skin response (GSR), and heart rate (HR) signals alongside behavioral contexts, demonstrating that fusing heterogeneous physiological data streams significantly enhances the classification robustness of the stress classification system. This direction is extended in the present work by introducing subject-level personalization via DT embeddings, enabling the model to adapt to inter-subject physiological variability without explicit post-training calibration. These findings collectively underscore the importance of joint multimodal signal processing in wearable stress detection and inform the 6-channel input design adopted in the proposed DT-GRU framework.

A fundamental limitation of population-level stress detection models is their failure to account for pronounced inter-subject physiological variability: the same external stressor elicits qualitatively different physiological patterns across individuals, depending on physical characteristics, prior stress exposure, and personality traits. While standard machine learning and deep learning architectures have established baseline performance on the multimodal WESAD dataset (Bobade and Vani, 2020), substantial performance degradation often occurs across unseen subjects due to this individual physiological heterogeneity, highlighting the distinct limitations of generic population-level models. Bolpagni et al. (2024) conducted a comprehensive scoping review of personalized stress detection frameworks, finding that biosignal-based personalization strategies, particularly individual calibration using subject-specific data, consistently improve classification performance across diverse sensing platforms. Similarly, Tutunji et al. (2023) demonstrated in a naturalistic study that individually tailored models significantly outperform generalized population-level frameworks under robust evaluation schemes.

Li et al (Li and Washington, 2024). further established that personalized models outperform generalized approaches across WESAD-based classification tasks, explicitly attributing performance gaps to inter-subject variability that subject-agnostic training inherently averages away. Despite these compelling findings, most personalization strategies require explicit per-subject fine-tuning or held-out calibration data, which is impractical in real-world deployment. The present work addresses this gap by encoding subject identity as a trainable latent embedding within the model architecture itself, eliminating the need for subject-specific post-training adaptation.

DT technology is increasingly used in healthcare as a paradigm for subject-specific, continuously updated virtual models that integrate physiological data to support monitoring and prediction (Vallée, 2023). Cellina et al. (2023) reviewed digital human twin applications in medicine and highlighted their potential to model complex multi-omics and physiological interactions to support individualized decision-making. Katsoulakis et al. (2024) further reported a growing trend toward incorporating wearable sensors into health DTs, particularly for continuous monitoring of chronic conditions. Sun et al. (2023) showed that coupling DTs with IoT wearables enables adaptive health monitoring by updating a subject’s virtual physiological profile from streaming sensor data. Despite this progress, the application of DT concepts to affective computing and physiological stress detection remains relatively underexplored. While existing healthcare DT frameworks focus predominantly on structural anatomy, cardiac physiology, or disease progression modeling, this work extends the digital twin paradigm to affective computing. We formalize a novel structural integration of trainable latent identity embeddings that act as continuous, subject-specific priors within a joint temporal-multimodal stress detection pipeline. The proposed DT-GRU framework addresses this gap by introducing a trainable subject-embedding module that serves as a compact parametric DT, capturing individual-level physiological characteristics within a unified end-to-end architecture.

## 3 Proposed approach

**Figure 1** illustrates the end-to-end pipeline of the proposed DT Enhanced Gated Recurrent Unit (DT-GRU) framework for wearable stress detection. The pipeline begins with dataset acquisition from the WESAD benchmark, where raw wrist-worn physiological signals comprising ACC, BVP, EDA, and TEMP are resampled to 4 Hz, fused into a six-channel representation, normalized per subject via Z-score standardization, and segmented into 30-second sliding windows. The aggregated windows are partitioned using a subject-independent train-test split, after which the dual-branch DT-GRU model processes each window through a stacked GRU temporal encoder in parallel with a trainable DT subject-embedding module, whose outputs are concatenated and passed through a fully connected sigmoid classifier for binary stress prediction.

**Figure 1:**
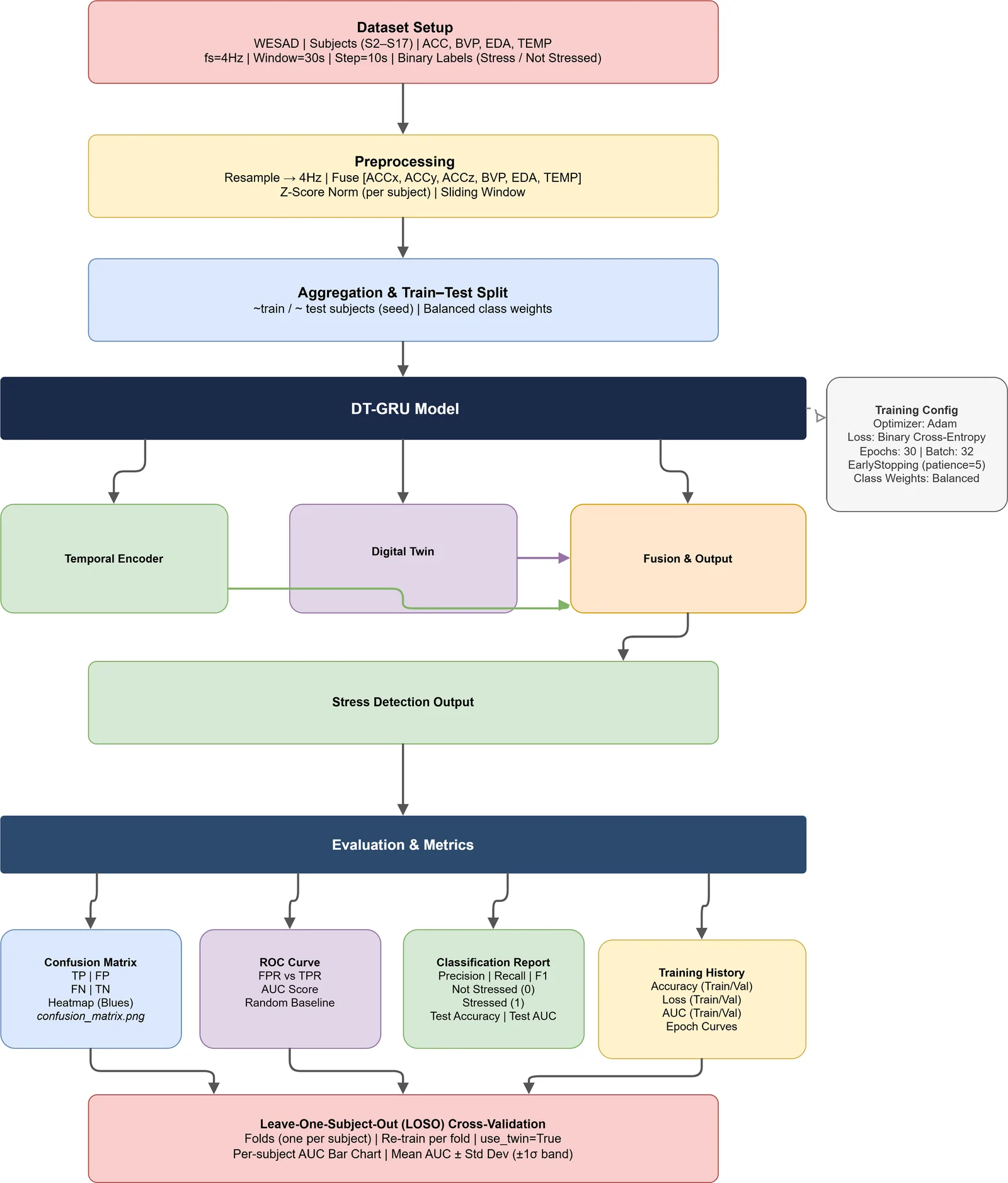
Digital twin enhanced GRU (DT-GRU) framework for wearable stress detection.

### 3.1 Dataset setup and configuration

**Algorithm 1** outlines the initialization procedure for the experimental pipeline. The WESAD dataset is first acquired programmatically, and a list of 15 valid subjects is defined by excluding participants S1 and S12, whose recordings are unavailable or incomplete. A binary label mapping M is then constructed to convert the original three-class affective state annotations into a two-class scheme, where baseline and amusement conditions are treated as non-stressed (class 0) and the stress condition is treated as stressed (class 1). The global signal processing parameters, including the target sampling frequency *f_s_*= 4 Hz, window length *W*_pts_ = 120 samples, and step size *S*_pts_ = 40 samples, are set once and applied uniformly across all subjects to ensure consistency throughout the pipeline. Finally, a subject-to-integer identity registry D is constructed, assigning each subject a unique integer index that is later used as input to the DT embedding module in Phase 5.

### 3.2 Subject-level data inspection

**Algorithm 2** describes the subject-level data inspection procedure carried out before any signal processing. For each subject in the dataset, the raw label array is extracted directly from the corresponding pickle file, in which labels were originally sampled at 700 Hz. The unique affective states present in each subject’s recording are identified, and for each state, the total sample count *C_l_*and the corresponding duration *D_l_* (in minutes) are computed. This analysis serves two important purposes. First, it confirms that each subject’s recording contains segments from both the stress and non-stress conditions, a fundamental prerequisite for binary classification. Second, it exposes the degree of inter-subject variability in affective state durations.

This key observation motivates the adoption of subject-aware evaluation strategies, particularly the Leave-One-Subject-Out cross-validation protocol used in the final experimental phase.

Algorithm 1

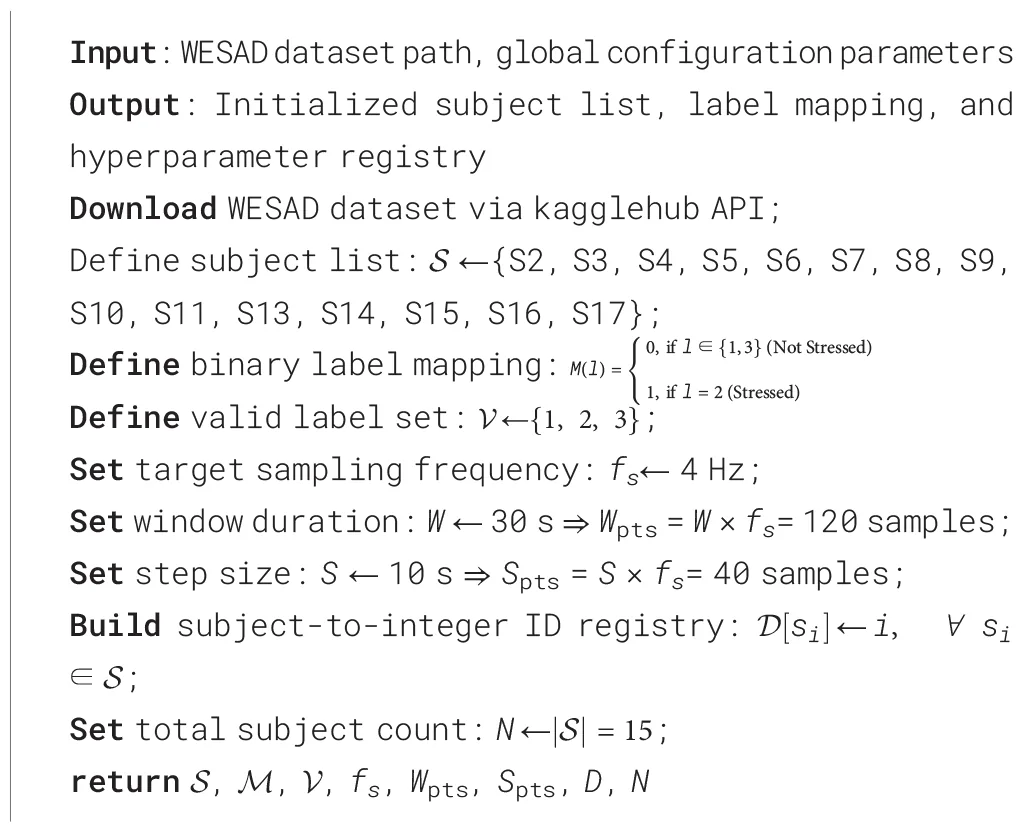

Algorithm 2

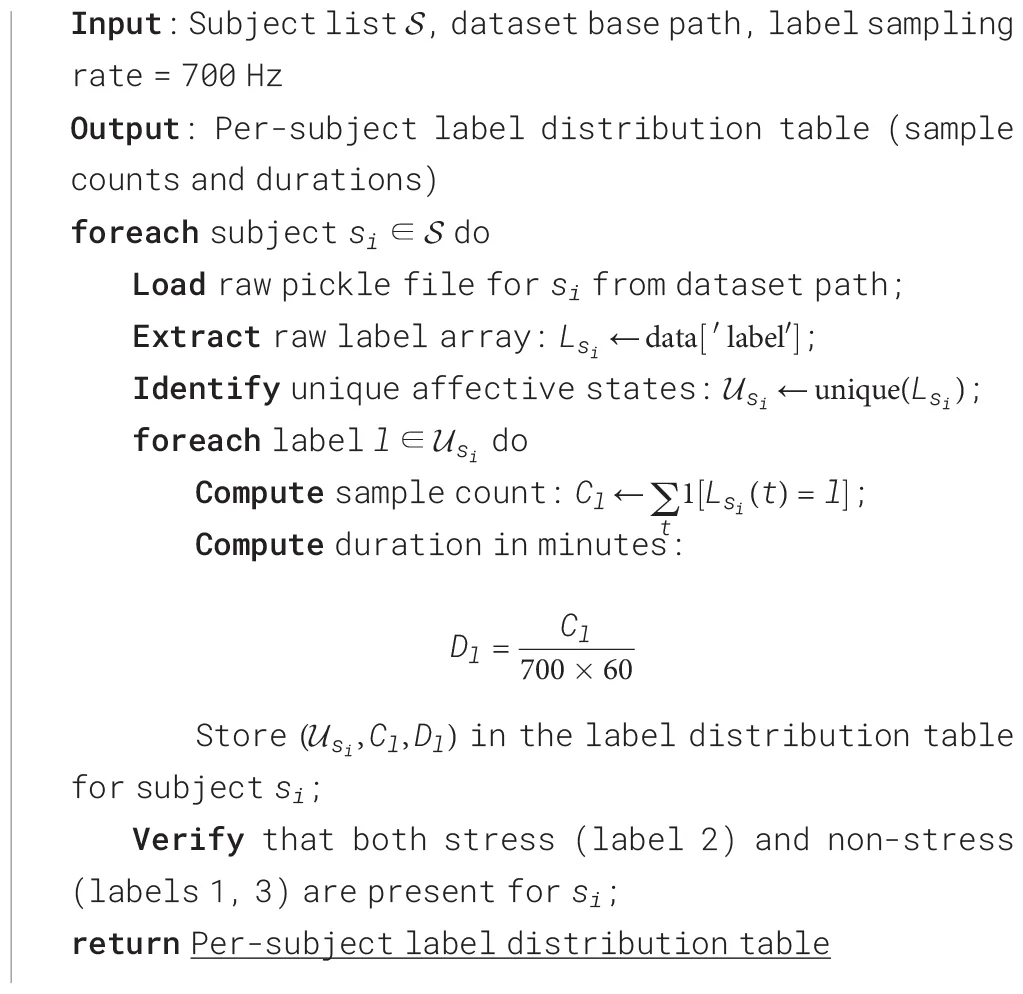

### 3.3 Preprocessing and window creation

**Algorithm 3** details the thoroughly executed processes of signal preprocessing and window creation. There are four steps in the process. In the first step, all wrist-worn physiological signals, which include three-axis accelerometry (ACC), blood volume pulse (BVP), electrodermal activity (EDA), and skin temperature (TEMP), are resampled from their native acquisition rates to a common target frequency of *f_s_*= 4*Hz* using Fourier-based resampling; the label array is downsampled and rounded accordingly to maintain temporal alignment. As the second step, we concatenate all resampled signals on the feature axis, obtaining one six-channel multivariate time series $X\in {\mathbb{R}}^{T_{\text{target}}\times 6}$ at every timestep, encoding the entire physiological state of the subject. In the third step, we apply per-subject Z-score normalization. For the Leave-One-Subject-Out (LOSO) cross-validation scheme, the StandardScaler is fit entirely on the held-out subject’s signal matrix to maintain cohort independence. Crucially, when evaluating with the standard subject-independent train-test split, the scaling parameters (*µ,σ*) are computed strictly from the designated training partitions and then applied to the test partitions to eliminate future data leakage. The last step of the procedure consists of partitioning the time series into windows of fixed length of *W*_pts_ = 120 samples (30 seconds) using a sliding window of step size *S*_pts_ = 40 samples (10 seconds). Based on the last timestamp, each window is assigned its class label. Further, only that window is retained whose label belongs to the set of valid affective states V={1,2,3}. A binary mapping ℳ is then applied to these retained windows to get the final classification targets.

Algorithm 3

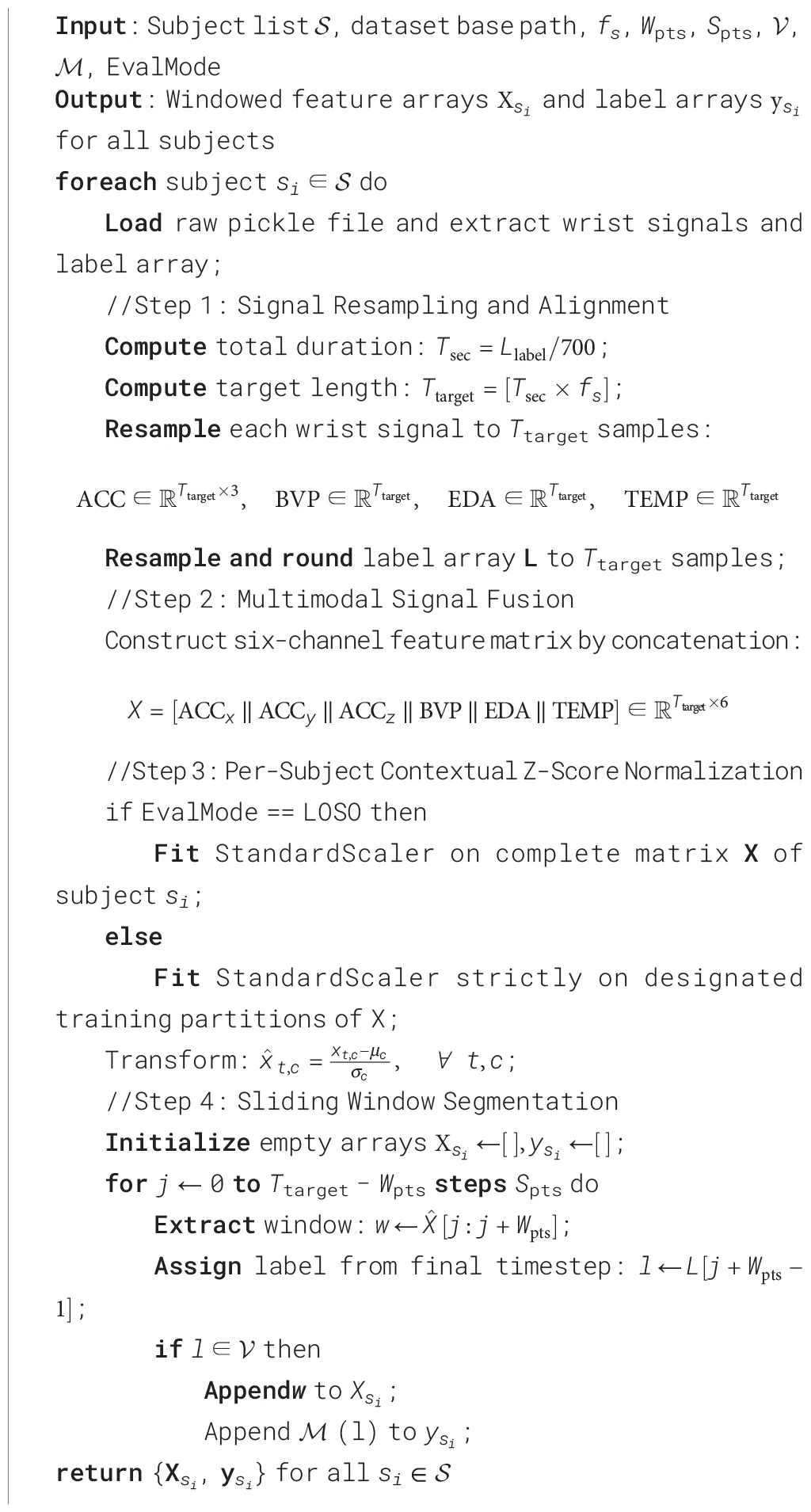

#### 3.3.1 Signal resampling and alignment

**Algorithm 4** explains how the initial signals gathered from the wrist are adjusted in terms of timing and sampling. The WESAD acquisition protocol has sampled the raw label array at 700 Hz. This information is first used to determine the total recording duration *T*_sec_. Thereafter, we obtain the target signal length *T*_target_ by multiplying *T*_sec_ with the desired output frequency *f_s_*= 4 Hz. Each of the four wrist-based modalities, three-axis accelerometry (ACC), blood volume pulse (BVP), electrodermal activity (EDA), and skin temperature (TEMP), is independently resampled to *T*_target_ samples using Fourier-based resampling via scipy.signal.resample. This method enables resampling of the signal in the Fourier domain while preserving the original signal’s spectral properties. Further, the temporal resolution is reduced. The label array is downsampled to *T_target_*and rounded to the nearest integer, similar to the signal. After resampling, all modalities are in the same temporal grid, so they can be directly concatenated into a single six-channel multivariate signal matrix $X\in {\mathbb{R}}^{T_{\text{target}}\times 6}$, where each row pertains to a given timestep, and each column to a given physiological channel. This time-aligned representation is the input to all subsequent preprocessing and windowing steps.

Algorithm 4

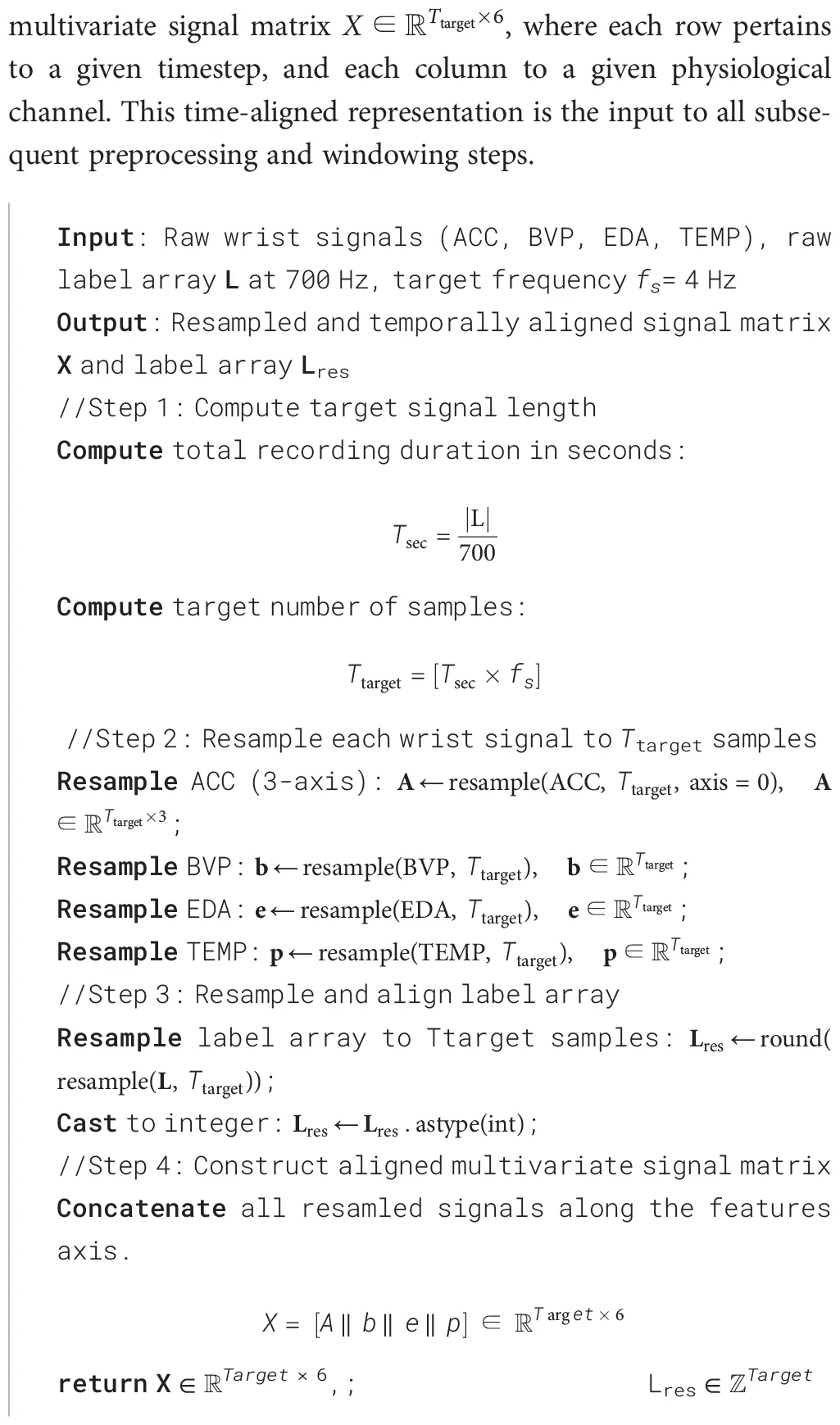

#### 3.3.2 Multimodal signal fusion

**Algorithm 5** describes the multimodal signal fusion procedure that combines the four resampled wrist-worn physiological modalities into a single unified feature representation. Following temporal alignment at the common sampling frequency of *f_s_*= 4 Hz, the three-axis accelerometry signal A is already a matrix of shape *T* × 3, while the remaining univariate signals BVP, EDA, and TEMP are each reshaped from one-dimensional vectors into column vectors of shape *T* × 1. All five components are then concatenated horizontally along the feature axis to produce the six-channel multivariate signal matrix $X\in {\mathbb{R}}^{T\times 6}$, where each row xt represents the complete physiological state of the subject at timestep t: $x_{t}={\left[{\text{ACC}}_{x},\;{\text{ACC}}_{y},\;{\text{ACC}}_{z},\text{ BVP},\text{ EDA},\text{ TEMP}\right]}^{⊤}\in {\mathbb{R}}^{6}$(1)

We use this fusion strategy because the different physiological modalities provide complementary insights into the autonomic stress response. This includes EDA, which captures sympathetic nervous system arousal via sweat gland activity; BVP, which encodes cardiovascular dynamics; TEMP, which captures changes in peripheral blood flow; and ACC, which provides motion context, allowing the model to disentangle stress-induced physiological changes from movement artifacts. The GRU temporal encoder in Phase 5 jointly models temporal dynamics and cross-channel dependencies within a unified sequential learning framework, representing all modalities as a single matrix.

Algorithm 5

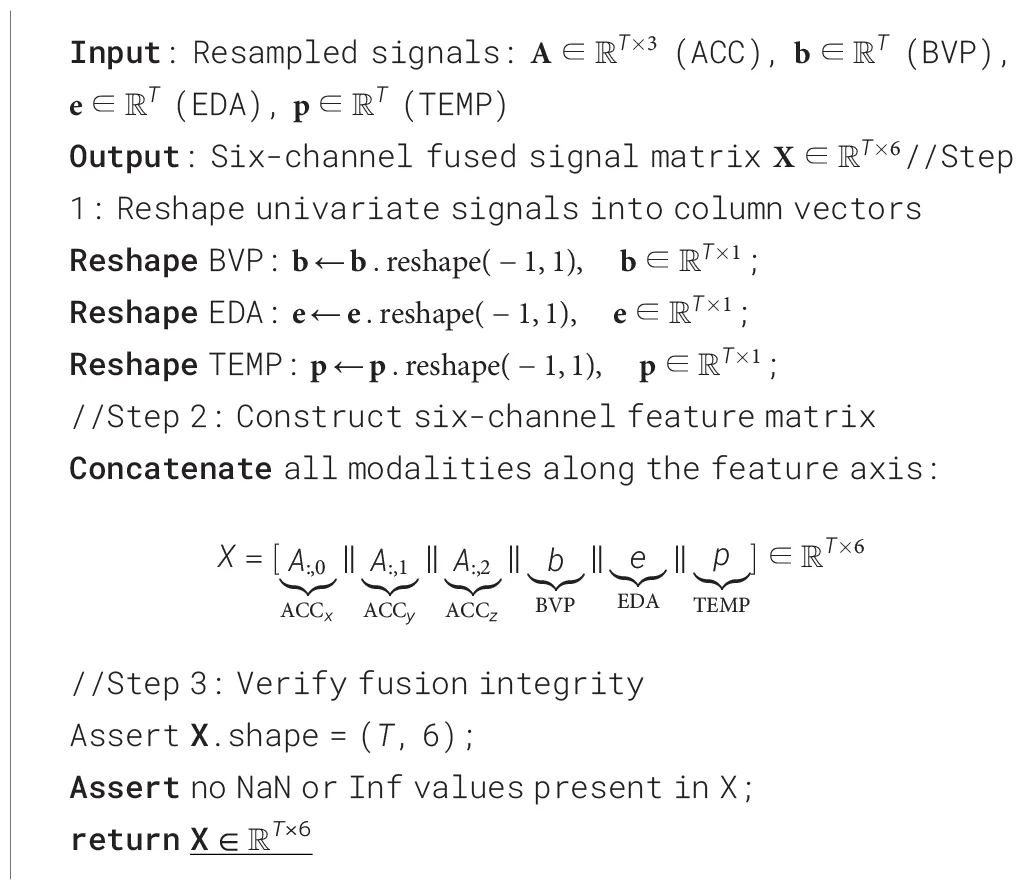

#### 3.3.3 Per-subject normalization

**Algorithm 6** shows that the Z-score normalization procedure is applied per subject on the six-channel fused signal matrix individually. Each physiological channel is assigned a channel mean and standard deviation computed uniquely from the signal of the current subject without reference to any other subject’s data. After this, each sample in the channel is centered by subtracting the channel mean and dividing by the channel standard deviation, yielding a zero mean and unit variance. Subject-level normalization will be preferred over global normalization for two reasons. The first benefit is that it removes inter-subject scale differences from natural physiological inter-subject variability; for instance, baseline EDA levels vary significantly across individuals. Thus, the subject’s stress-related model learns only deviations from the subject’s baseline, not the average. The second important point is that it avoids data leakage by ensuring that the normalization of training subjects does not use statistical information from test subjects. Otherwise, it will artificially inflate evaluation performance and create unrealistically optimistic results. The normalized matrix is of size suggested $\hat{X}\in {\mathbb{R}}^{T\times 6}$ and is passed directly to the sliding window segmentation step.

#### 3.3.4 Sliding window segmentation

**Algorithm 7** presents the sliding window segmentation procedure applied to each subject’s normalized multivariate signal matrix. We partition the normalized time series $\hat{X}\in {\mathbb{R}}^{T\times 6}$ into a sequence of fixed-length overlapping windows using a window length of *W*_pts_ = 120 samples, corresponding to 30 seconds of physiological data at *f_s_*= 4 Hz, and a step size of *S*_pts_ = 40 samples, corresponding to a 10-second.

Algorithm 6

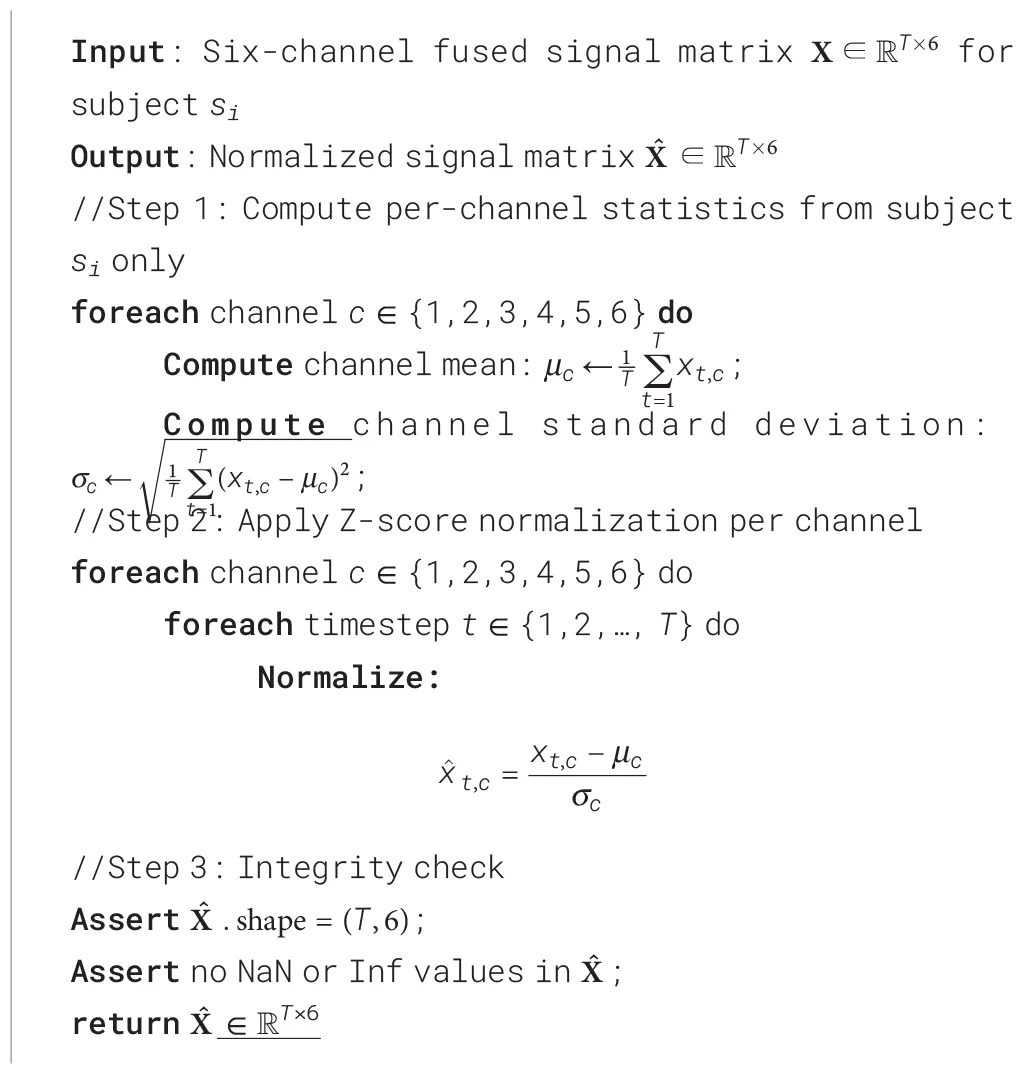

shift between consecutive windows. The sliding window approach with overlapping steps is deliberately adopted to maximize the number of training samples extracted from the limited per-subject recordings, while the 30-second window duration is chosen to capture sufficient temporal context for the GRU encoder to model meaningful stress-related physiological dynamics. The class label for each window is assigned from the label at the final timestep of that window, consistent with the standard practice of treating physiological windows as reflecting the affective state at the end of the observation interval. Windows whose final-timestep label does not belong to the valid affective state set V={1,2,3} corresponding to transient or unlabeled recording periods are discarded to ensure label integrity. For retained windows, the binary mapping ℳ is applied to convert the original multi-class label into a binary stress classification target, yielding the final windowed dataset $X_{s_{i}}\in {\mathbb{R}}^{N_{w}\times 120\times 6}$ and the corresponding binary label vector $y_{s_{i}}\in {\left\{0,1\right\}}^{N_{w}}$ for subject *s_i_*.

### 3.4 Dataset aggregation and train-test split

**Algorithm 8** outlines the process for aggregating datasets. The initial phase involves concatenating the windowed feature arrays and binary label vectors generated for each subject into three global arrays. These arrays include a feature tensor denoted as Xall, a binary label vector denoted as yall, and a subject identity vector denoted as sall. Here, *N* refers to the aggregate of windows among all 15 subjects. The vector identity of the subject is important because it will serve as direct input to the DT embedding module in phase 5 and help the model align each window to the subject-relevant latent representation. The second step uses a randomly chosen subject-independent train-test split. Specifically, about one-third of the subjects (approximately *n*_test_ ≈ 5) are chosen for the test set. A fixed random seed of 42 is used for reproducibility. The remaining subjects (approximately *n*_train_ ≈ 10) form the training set. This protocol guarantees that.

Algorithm 7

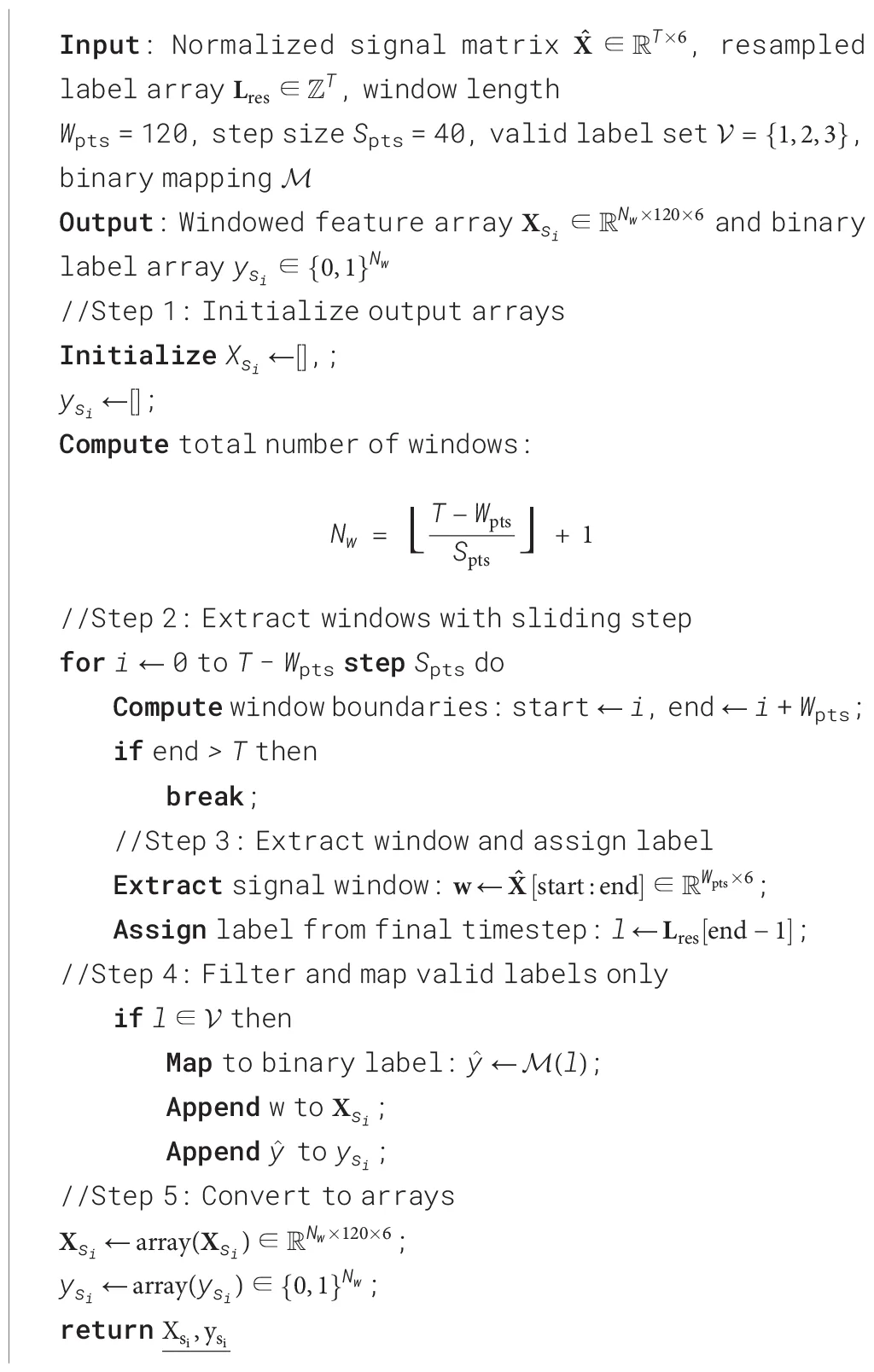

the subjects found in the training and evaluation data are distinct, enabling a more realistic evaluation of model performance on unseen data. In the last step, the class weights are calculated based on the training label distribution to mitigate the inherent class imbalance between stress and non-stress windows in the WESAD dataset. This imbalance is intimately linked to the annotation used to create the dataset. The weight assigned to class *c* is inversely proportional to the frequency of class *c* in the training set. During model training, we apply these weights to penalize misclassification of the minority stress class and to avoid creating a bias toward the majority non-stress class.

### 3.5 GRU model with DT embedding and training

The proposed DT-GRU model is shown in **Algorithm 9**. It enables simultaneous application to two inputs: the physiological signal window and the subject-identity scalar. The temporal encoder employs two stacked GRU layers with 64 and 32 units, respectively, to sequentially encode the signal from a sliding input window. In this case, $h_{T}\in {\mathbb{R}}^{32}$ captures the stress-related temporal dynamics of the signal. At the same time, the DT module uses the integer subject identity to define a trainable embedding vector $e_{s}\in {\mathbb{R}}^{16}$ which acts as the latent physiological profile of each subject. The 2 representations are concatenated to obtain a 48-D vector. This is then fed into a Dense layer with ReLU activation and Dropout. Then, the.

Algortihm 8

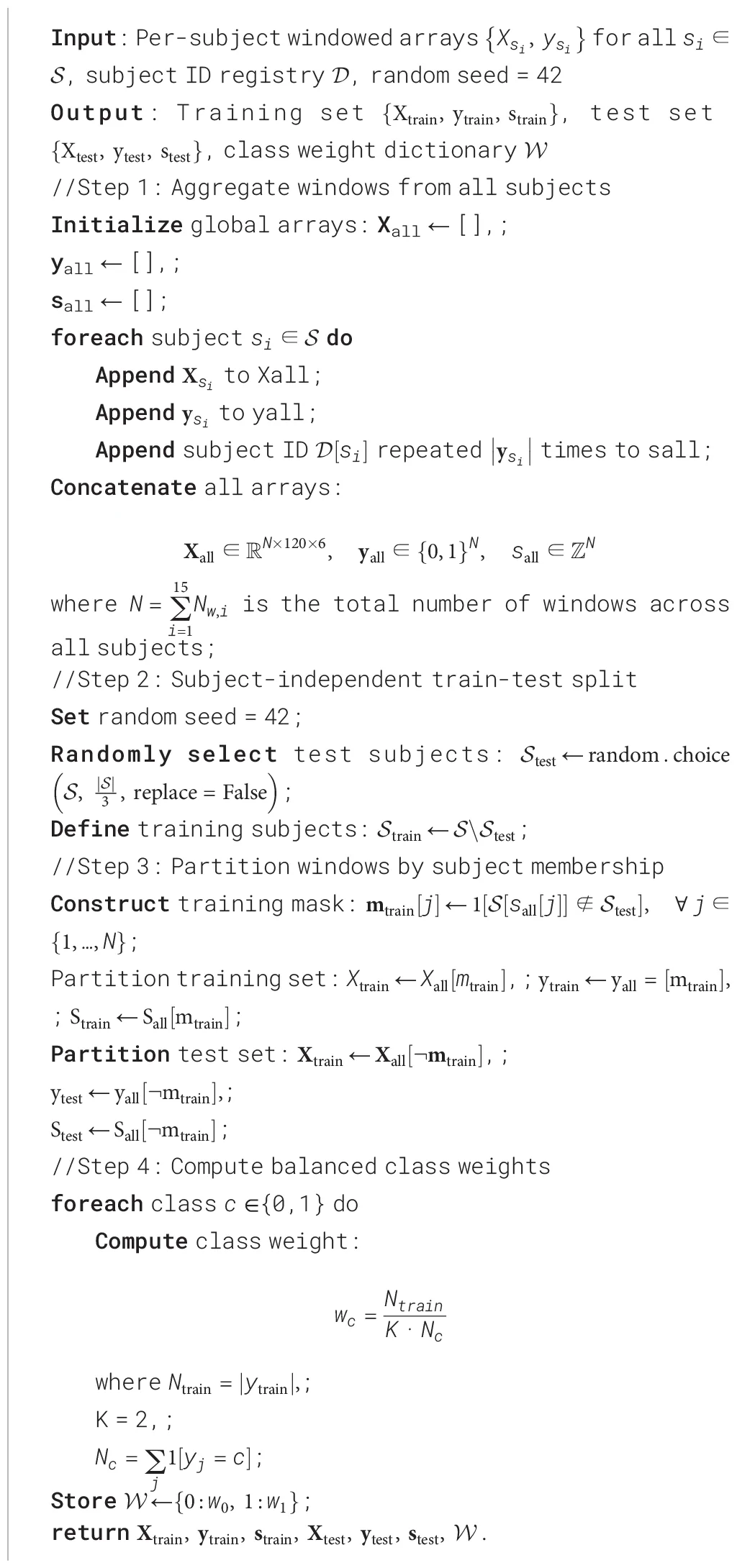

layer outputs a scalar via a sigmoid to classify stress as binary. For training, we use the Adam optimizer with binary cross-entropy loss and balanced class weights. An early stopping callback prevents overfitting.

Algorithm 9

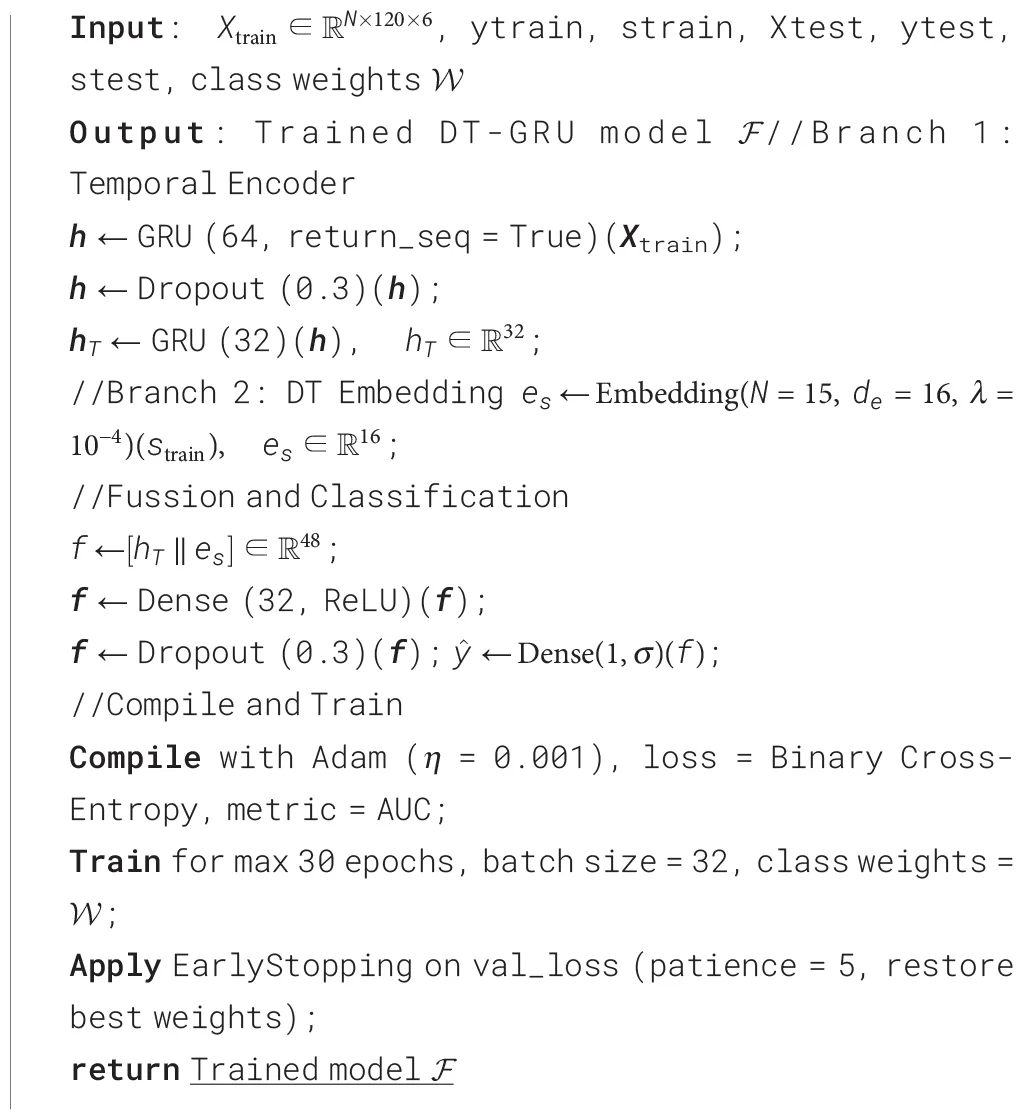

#### 3.5.1 Temporal encoder

The temporal encoder, defined in **Algorithm 10**, extracts a compact sequential representation from the input physiological signal window. We send the 120-timestep, six-channel signal window $X_{w}\in {\mathbb{R}}^{120\times 6}$ through a GRU layer of 64 units with returnsequences=True. This layer generates the hidden state sequence $H\in {\mathbb{R}}^{120\times 64}$. This sequence preserves the temporal evolution of physiological features across all timesteps. To combat overfitting, a Dropout layer with a rate of 0.3 is used in the sequence. The resulting output from the regularized sequence is then fed to a second GRU layer. Moreover, this second GRU has 32 units with returnsequences=False. The second GRU provides just the final hidden state $h_{T}\in {\mathbb{R}}^{32}$ as a fixed-length summary of the entire 30-second physiological window. The two-layer GRU stack design allows the first layer to capture low-level temporal patterns for analysis, while the second layer models higher-level patterns. This provides a rich yet compact representation that is compatible with the DT subject embedding used for stress classification.

Algorithm 10

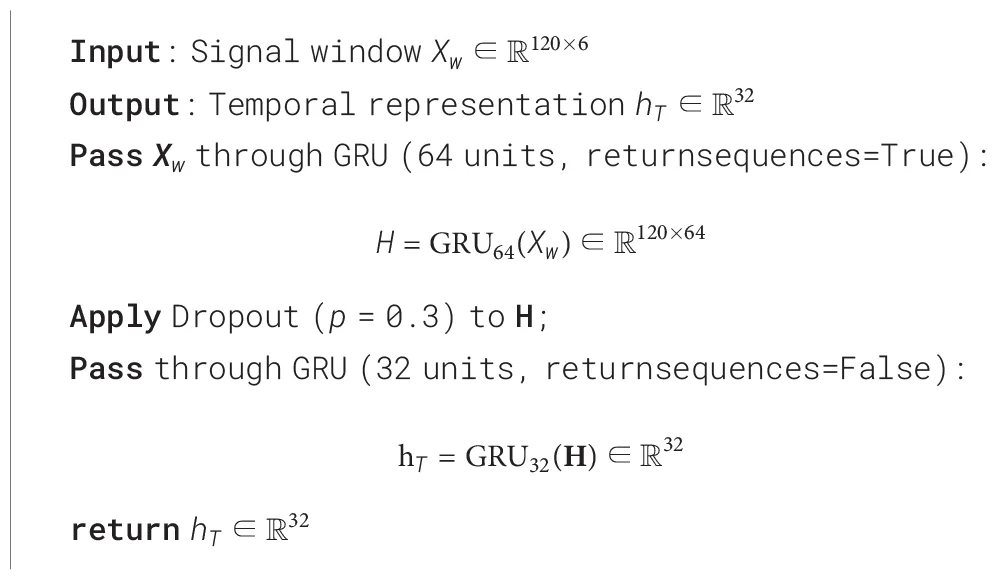

#### 3.5.2 Digital Twin embedding module

The DT embedding module is detailed in **Algorithm 11** and encodes each subject’s identity as a learnable, low-dimensional vector that serves as a compact latent representation of the subject’s physiology. An embedding matrix $\text{E}\in {\mathbb{R}}^{15\times 16}$ is initialized to encode the subjects, where each row E[s] corresponds to one of the 15 subjects. Each column is a learned latent physiological dimension. The module performs a simple look-up operation on the integer subject identity s and yields the embedding vector $e_{s}\in {\mathbb{R}}^{16}$. The embedding is not engineered by hand but is updated end-to-end during model training via backpropagation, thereby enabling it to encode the patient-specific physiological characteristics most predictive of stress. To prevent the embeddings for each subject from fitting to the subject-specific noise, we add L2 regularization on the embedding weights with *λ* = 10^−4^. The vector **e***_s_*corresponds to the parametric DT, a personalized physiological prior, that is combined with the output from the temporal encoder in the next fusion stage to condition the stress prediction on the identity of the person being assessed.

Algorithm 11

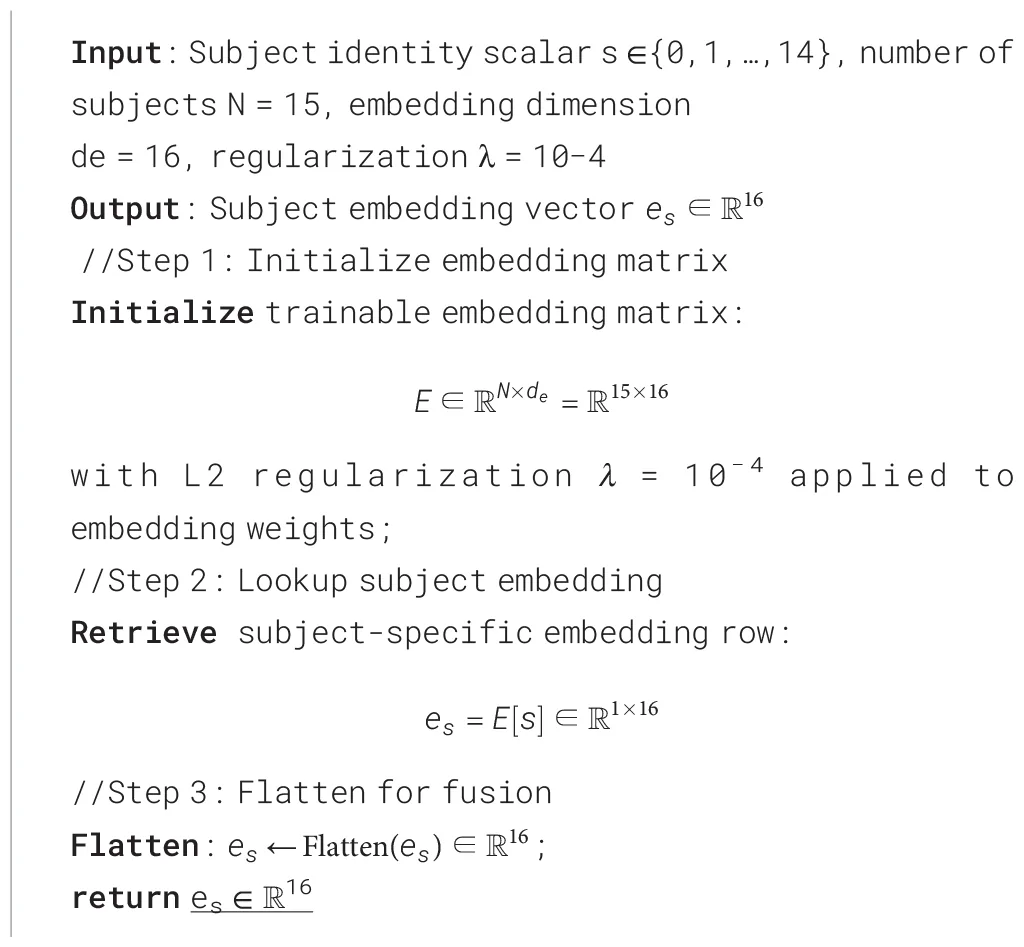

#### 3.5.3 Fusion and classification head

**Algorithm 12** presents the fusion and classification head, which combines the outputs of the temporal encoder and the DT embedding module to produce the final binary stress prediction. The 32-dimensional temporal representation hT, encoding the sequential physiological dynamics of the input window, and the 16-dimensional subject embedding es, encoding the individual physiological identity of the subject, are concatenated along the feature axis to form a 48-dimensional fused representation $f\in {\mathbb{R}}^{48}$. This concatenation-based fusion strategy is adopted because it preserves the full information content of both branches without compression, allowing the subsequent Dense layer to learn flexible interactions between temporal signal features and subject-specific characteristics. The fused vector is then projected through a fully connected Dense layer of 32 units with ReLU activation, which learns a non-linear combination of the temporal and subject-level features most discriminative for binary stress classification. A Dropout layer with rate p = 0.3 is applied after the Dense layer to reduce overfitting. Finally, a single-unit output layer with sigmoid activation maps the 32-dimensional representation to a scalar probability $\hat{y}\in \left[0,1\right]$, which is thresholded at 0.5 to produce the binary class prediction $\tilde{y}$, where $\tilde{y}=1$ indicates a stressed state and $\tilde{y}=0$ indicates a non-stressed state.

Algorithm 12

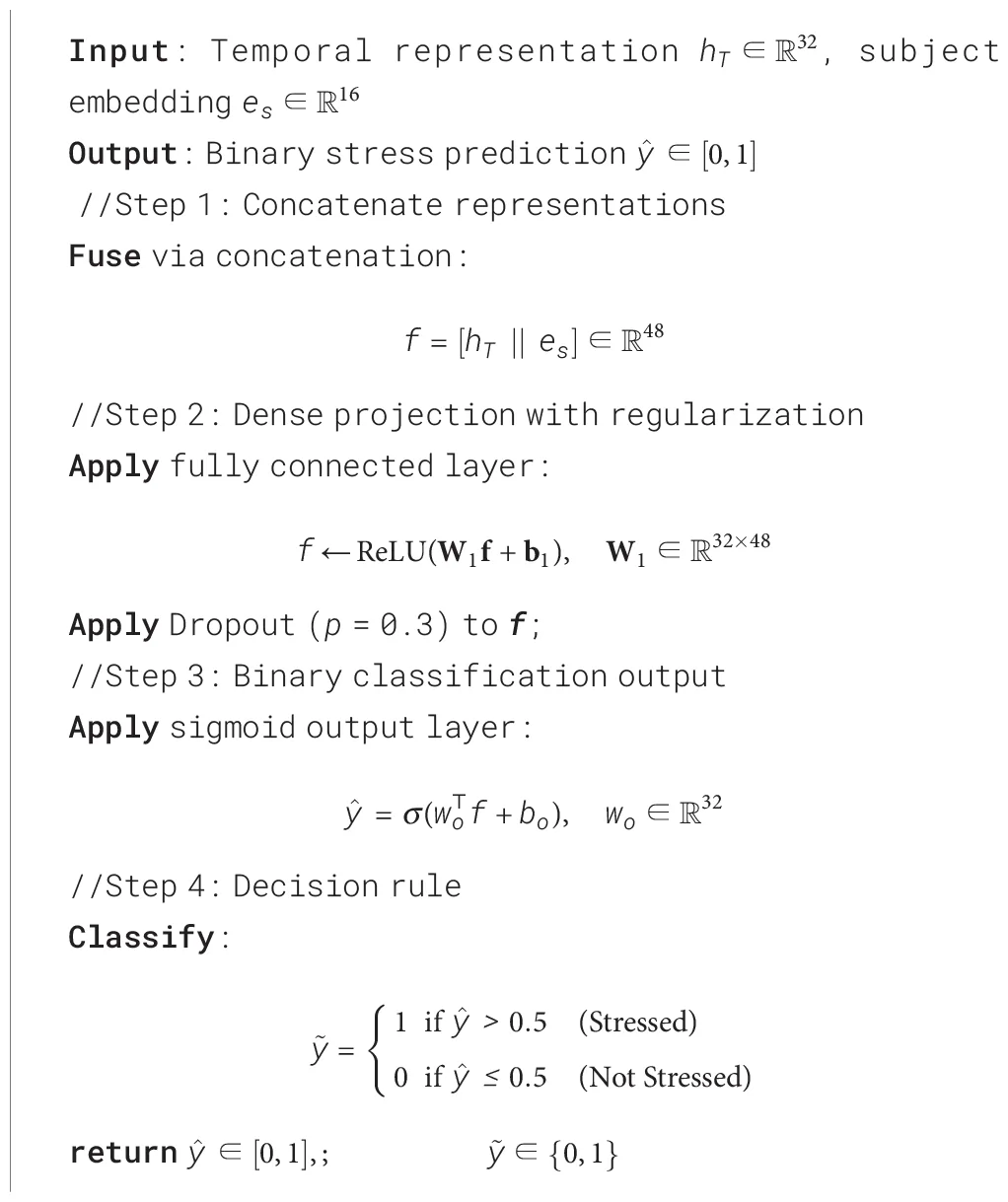

#### 3.5.4 Model compilation and training

**Algorithm 13** provides the methods for constructing the DT-GRU model along with its training procedure. The Adam optimizer (*η* = 0.001), which employs adaptive gradient-based parameter updates and is well known for converging well on deep learning models trained on physiological time-series data, is used to compile the model. The loss is weighted binary cross-entropy, in which the pre-computed class weights W are incorporated directly into the loss to penalize misclassification of the minority stress class in proportion to its under-representation in the training set. During the training process, evaluation metrics such as the classification accuracy and the area under the ROC curve AUC are monitored. The model is trained for up to 30 epochs, with a mini-batch size of 32 windows. An early stopping callback tracks the validation loss at the end of each epoch, stopping training if no improvement is observed after 5 epochs and automatically restoring the weights to the epoch with the lowest validation loss. This method avoids overfitting to the training subjects and ensures that the final model corresponds to the best-performing architecture observed during training.

### 3.6 Evaluation and metrics

**Algorithm 14** describes the evaluation protocol used to evaluate the trained DT-GRU model on held-out test subjects. The model produces scores $\hat{y}\in \left[0,1\right]$ for each test window resulting in a predicted class $\tilde{y}$, equal to 1 when $\hat{y}>0.5$. To measure the model’s classification performance, we compute 5 standard metrics. Accuracy is the overall proportion of correctly classified windows for both the negative and positive classes. Precision measures the proportion of predicted stressed windows that are actually stressed,

Algorithm 13

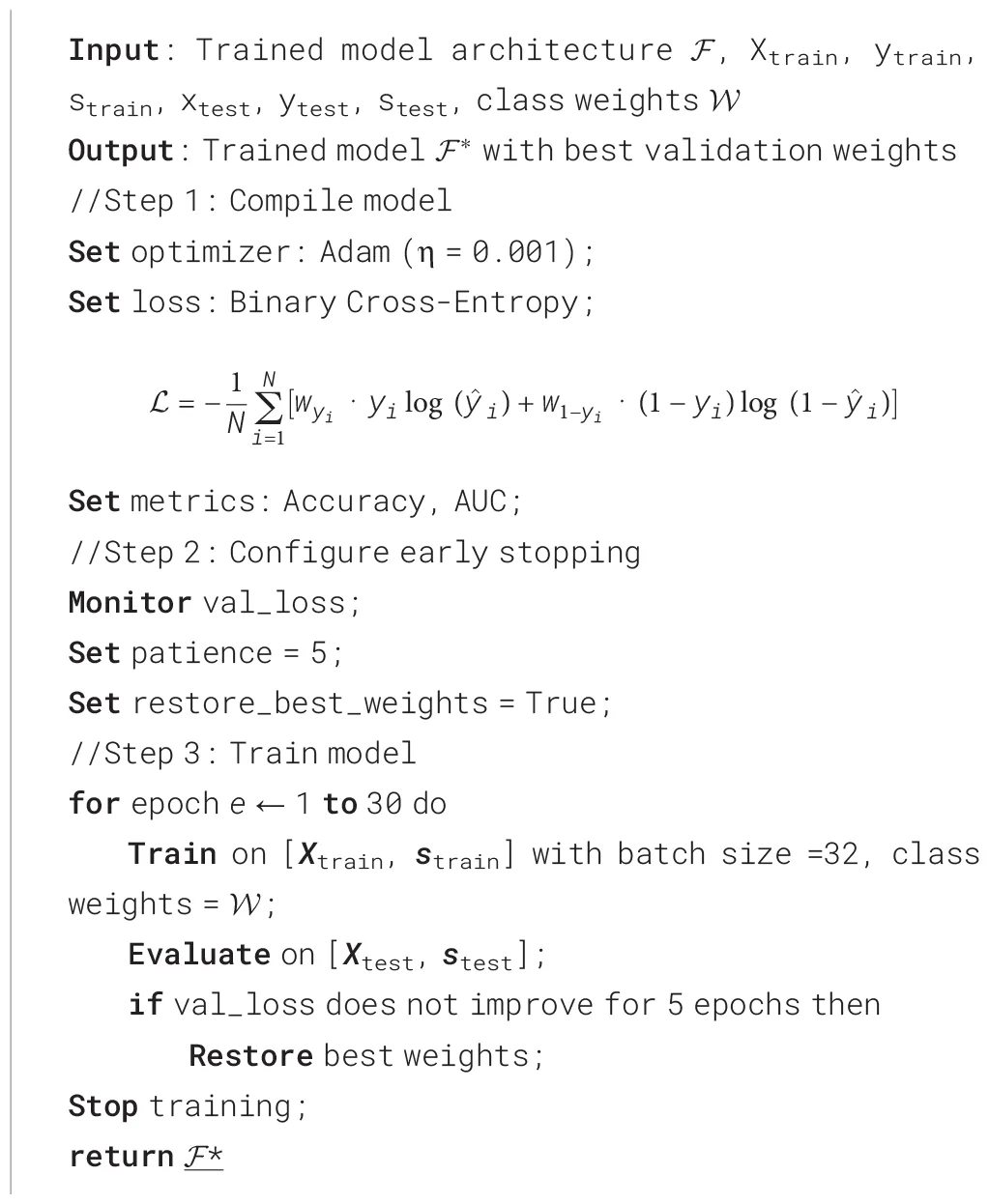

whereas Recall measures the percentage of actual stressed windows that the model correctly predicts. The F1-Score is described in terms of the harmonic mean of Precision and Recall. It combines the two metrics to provide a single Figure that is particularly informative when class imbalance is present. The Area under the ROC Curve (AUC) is calculated as the first metric; it quantifies a model’s discriminative ability across all classification thresholds. In addition to these scalar metrics, training vs. validation curves for accuracy, loss, and AUC are plotted to assess convergence behavior and any overfitting. Additionally, a confusion matrix is generated to inspect the distributions of true positives, true negatives, false positives, and false negatives. Finally, the ROC curve is plotted to illustrate the true positive rate vs. false positive rate trade-off across all decision thresholds.

### 3.7 Leave-One-Subject-Out experiment

**Algorithm 15** represents the LOSO (Leave-One-Subject-Out) cross-validation procedure that is used to evaluate the cross-subject generalizability of the proposed DT-GRU framework. Within each of the 15 folds, data from a single subject is fully held out as the test set while the other 14 subjects comprise the training set. In every fold, we initialize and train a fresh instance of the DT-GRU model from scratch to ensure that information from the test subject does not influence the model parameters. To prevent overfitting in each fold, early stopping is applied to the training loss with a patience of 3 epochs. The main evaluation metric selected for the LOSO experiment is the Area Under the ROC Curve (AUC), as it is robust to class imbalance and can be used to evaluate the discriminative performance independent of the decision threshold. When all 15 folds are complete, we obtain the per-subject AUC. We compute the mean AUC and standard deviation across all subjects. A bar chart is created of per-subject AUC scores along with the mean AUC line and a ±1 standard deviation band to show the subject-level variability in cross-subject.

Algorithm 14

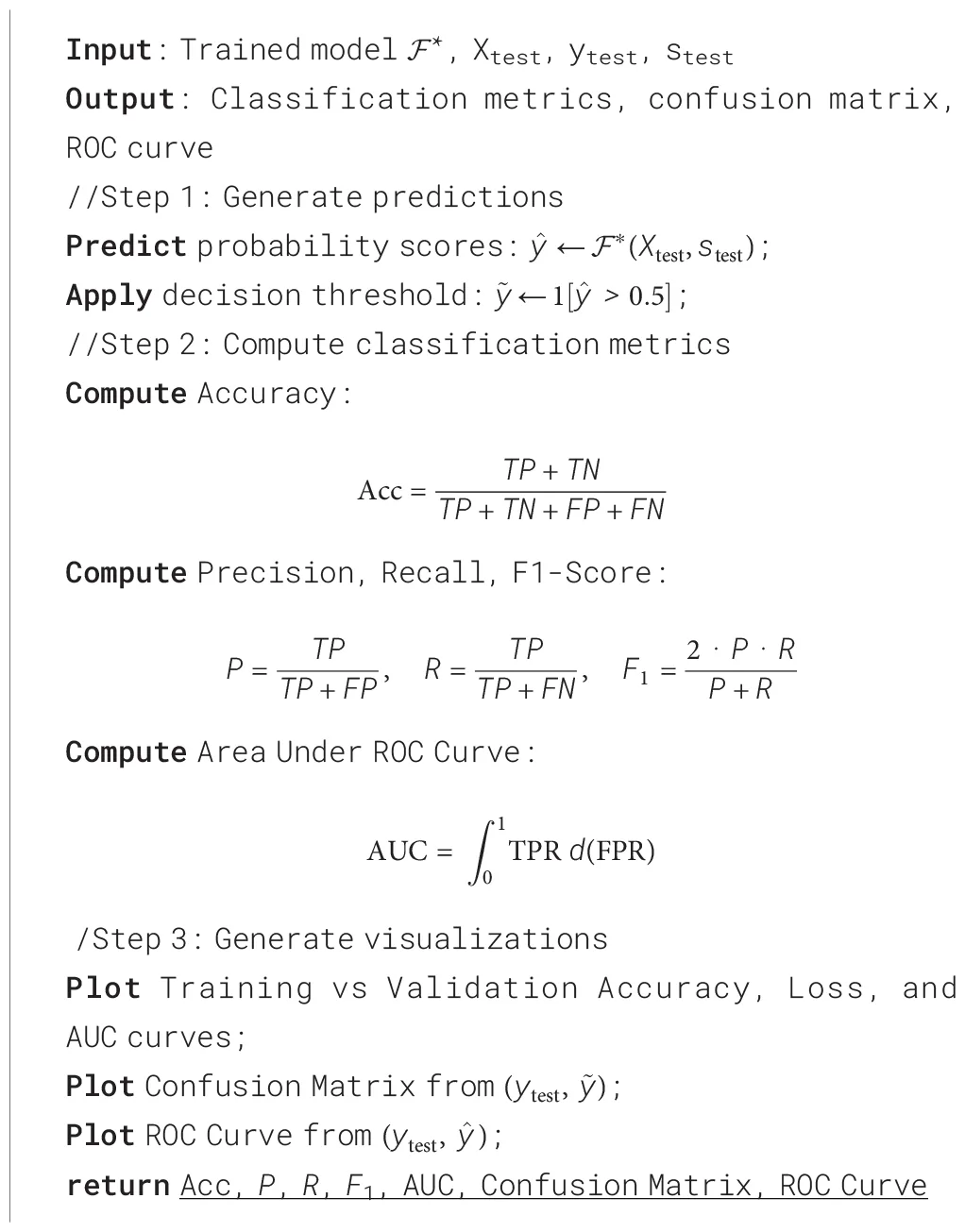

generalization. This comprehensive evaluation methodology is necessary to demonstrate the practical deployability of the proposed framework in wearable stress-monitoring scenarios, where models must generically classify physiological data from unseen subjects during training.

## 4 Results and discussion

### 4.1 Training performance

**Table 1** presents the architecture and parameter summary of the proposed DT-GRU model as generated from the model summary output. The complete model has 25,073 trainable parameters, requires 97.94 KB of memory, and contains no non-trainable parameters; all components, including the DT subject embedding, are optimized end-to-end during training.

**Table 1:** DT-GRU model parameter summary.

Parameter type	Count (memory)
Total Parameters	25,073 (97.94 KB)
Trainable Parameters	25,073 (97.94 KB)
Non-trainable Parameters	0 (0.00 B)

The model’s lightweight nature, with fewer than 26,000 trainable parameters, confirms that the DT-GRU framework is computationally efficient and well-suited for deployment in resource-constrained wearable sensing environments. Despite its compact size, the model achieves strong classification performance, as reported in Section 4.2, demonstrating that the DT embedding enables effective subject-level personalization without substantially increasing model complexity.

**Figure 2** presents the training and validation performance of the proposed DT-GRU model over 7 epochs (0-6) across three metrics: accuracy, loss, and AUC.

**Figure 2:**
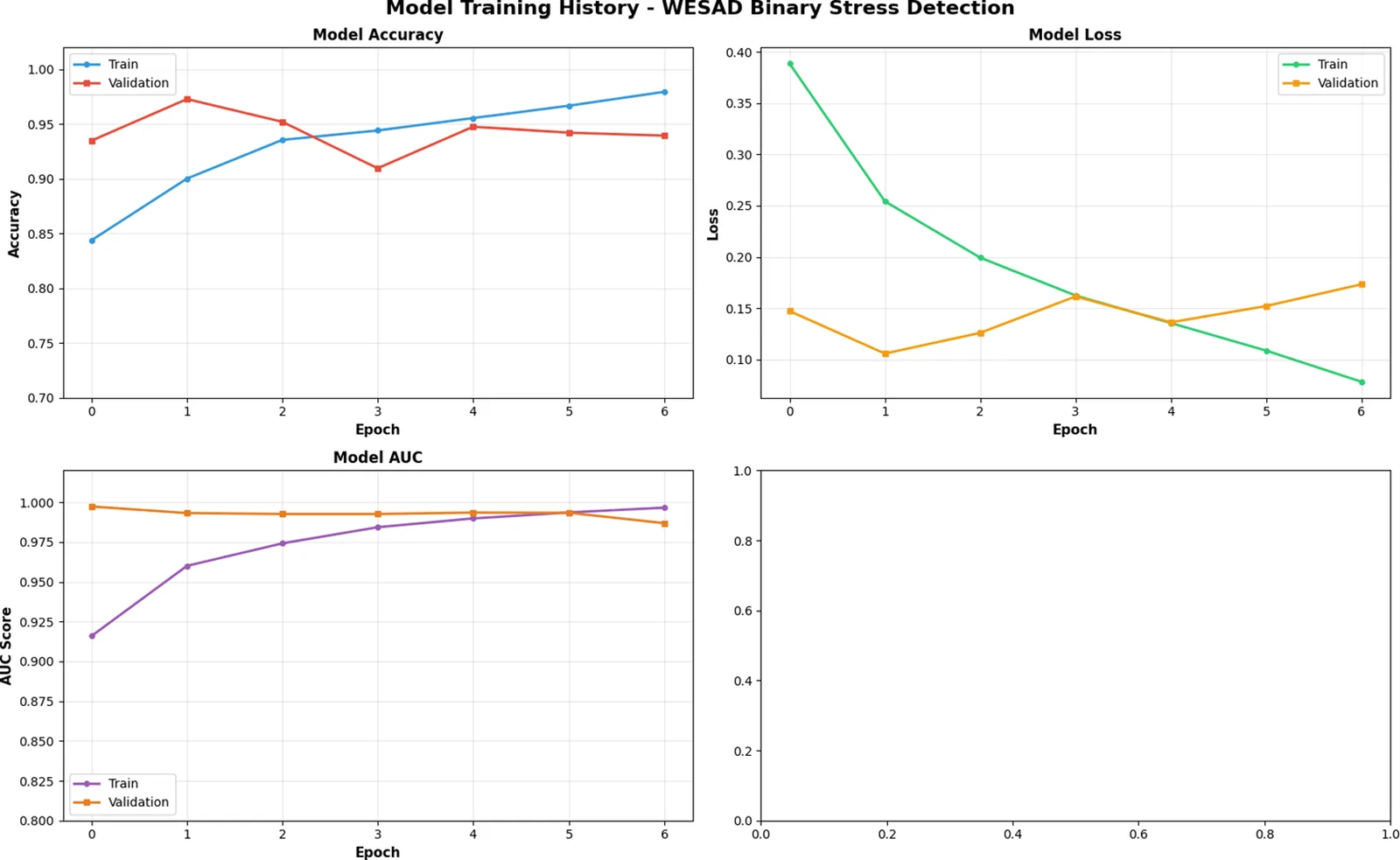
Training and validation performance curves of the DT-GRU model over 7 epochs on the WESAD binary stress detection task. Top-left: Model Accuracy. Top-right: Model Loss. Bottom-left: Model AUC. The bottom-right subplot is reserved for the ROC curve reported in Section 4.2.

Model Accuracy (Top-Left): The training accuracy (blue) is 0.84 at epoch 0 and increases steadily to 0.98 at epoch 6. The model is learning well across training subjects. The validation accuracy (red) starts at.

Algorithm 15

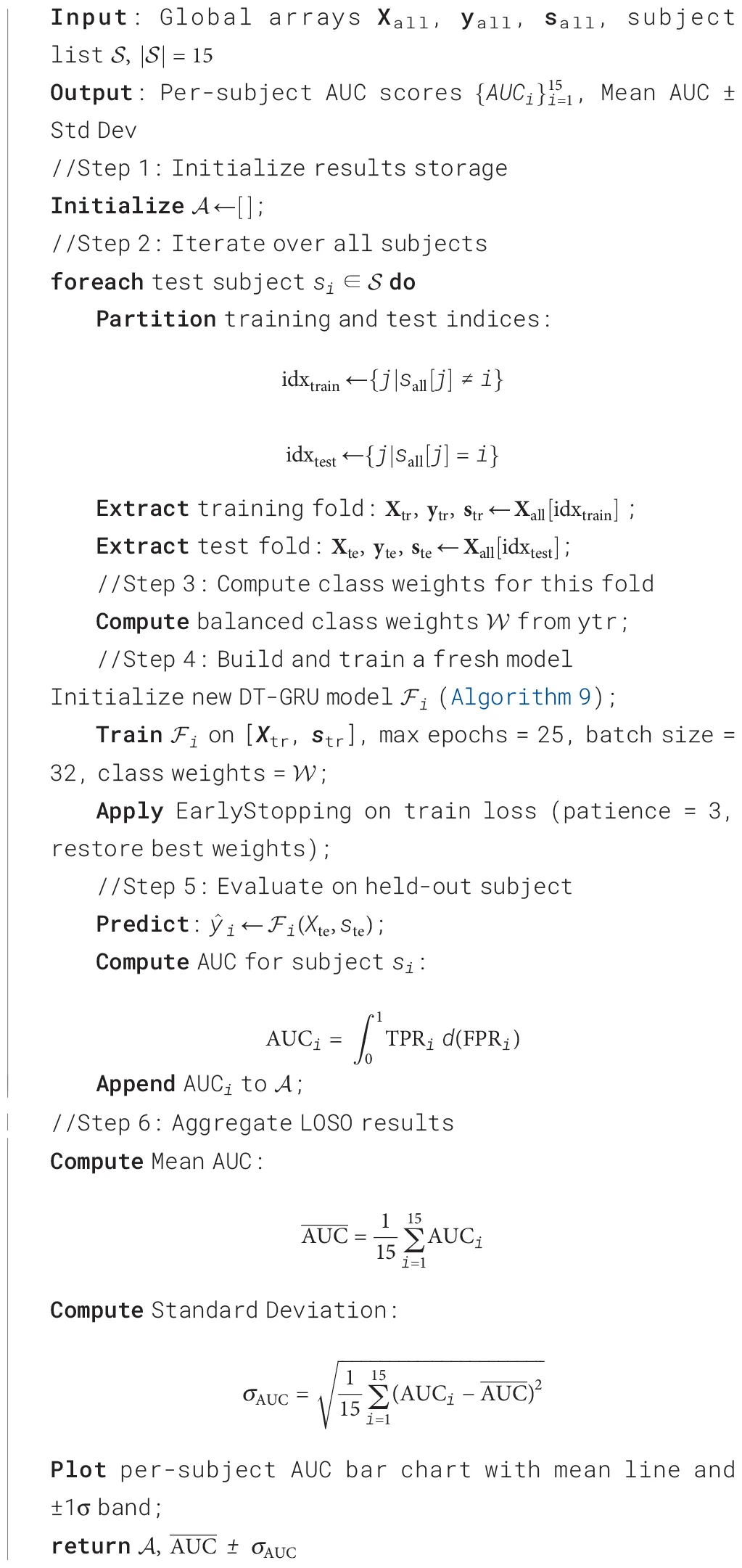

0.93 and reaches 0.97 at epoch 1. Afterward, the validation accuracy (red) fluctuates between 0.91 and 0.95 across the remaining epochs. The model can make correct predictions even at the early stage of training. As shown in the upper right corner of the image, the model loss (green) decreases from 0.39 at epoch 0 to 0.08 at epoch 6. The model was observed to be trainable and convergent. The validation loss (orange) reaches its first peak at epoch 1 (approximately 0.10) and gradually increases to 0.17 at epoch 6. This diverges from training, and the validation loss after epoch 1 indicates mild overfitting to the training subjects, which the early stopping callback mitigates by restoring the best weights from epoch 1.

Model AUC (Bottom-Left): The training AUC (purple) steadily improves from 0.92 at epoch 0 to 0.99 by epoch 6. The validation AUC (orange) remains consistently high throughout training, starting at 0.997 at epoch 0, and stabilizes between 0.985 and 0.995 across all other epochs. In the early epochs, the validation AUC consistently remains higher than the training AUC, implying that the DT subject embeddings rapidly encode subject-specific physiological traits. Therefore, even in the early phases of training, the embeddings demonstrate strong discriminative performance on the held-out subjects.

### 4.2 Classification results

**Table 2** presents the six evaluation metrics of the proposed DT-GRU model on the held-out test set, comprising 1,107 windows from the subject-independent test subjects.

**Table 2:** Classification performance of the proposed DT-GRU model on the test set.

Class	Precision	Recall	F1-Score	Support
Not Stressed	0.99	0.98	0.98	772
Stressed	0.94	0.97	0.96	335
Accuracy		0.97		1107
Macro Avg	0.97	0.97	0.97	1107
Weighted Avg	0.97	0.97	0.97	1107
Test Accuracy: 97.29%		Test AUC: 0.9931		

The DT-GRU model achieves 97.29% test accuracy and 0.9931 test AUC on the external test set (1107 windows). The model achieves 0.99 precision for the Not Stressed class, meaning that 99% of the windows.

predicted as Not Stressed are, in fact, Not Stressed. It also has 0.98 recall, meaning 98% of all non-stressed windows are correctly predicted. F1-score for the Not Stressed class is 0.98, from 772 test windows. The model’s precision and recall for the Stressed class are 0.94 and 0.97, respectively, yielding an F1-score of 0.96 across 335 test windows. The precision of the Stressed class is low due to moderate class imbalance. The classification errors primarily involved mislabeling some stressed windows as non-stressed, though the number was limited. With a recall of 0.97 for the Stressed class, the model detects 97% of actual stress events. The overall precision, recall, and F1-score (macro- and weighted-averaged) are 0.97, indicating balanced performance and consistent predictability across classes.

**Figure 3** presents the confusion matrix of the proposed DT-GRU model, which was evaluated on the held-out test set of 1,107 windows. Out of the 772 true Not Stressed windows in our real data, the model correctly classifies 753 as Not Stressed (true negative) and misclassifies only 19 as Stressed (false positive). Of the 335 Stressed windows, 324 are correctly classified as Stressed by the model (true positives), while 11 are misclassified as Not Stressed (false negatives). The results indicate that the model makes only a few mistakes either way. According to the proposed framework, the false-negative rate of 11 is clinically significant, as it translates to fewer than 3.3% of true stress episodes being missed. This demonstrates the dependability of wearable systems for stress monitoring. The 19 false positives mean the model rarely raises stress alerts for people who are not really stressed.

**Figure 3:**
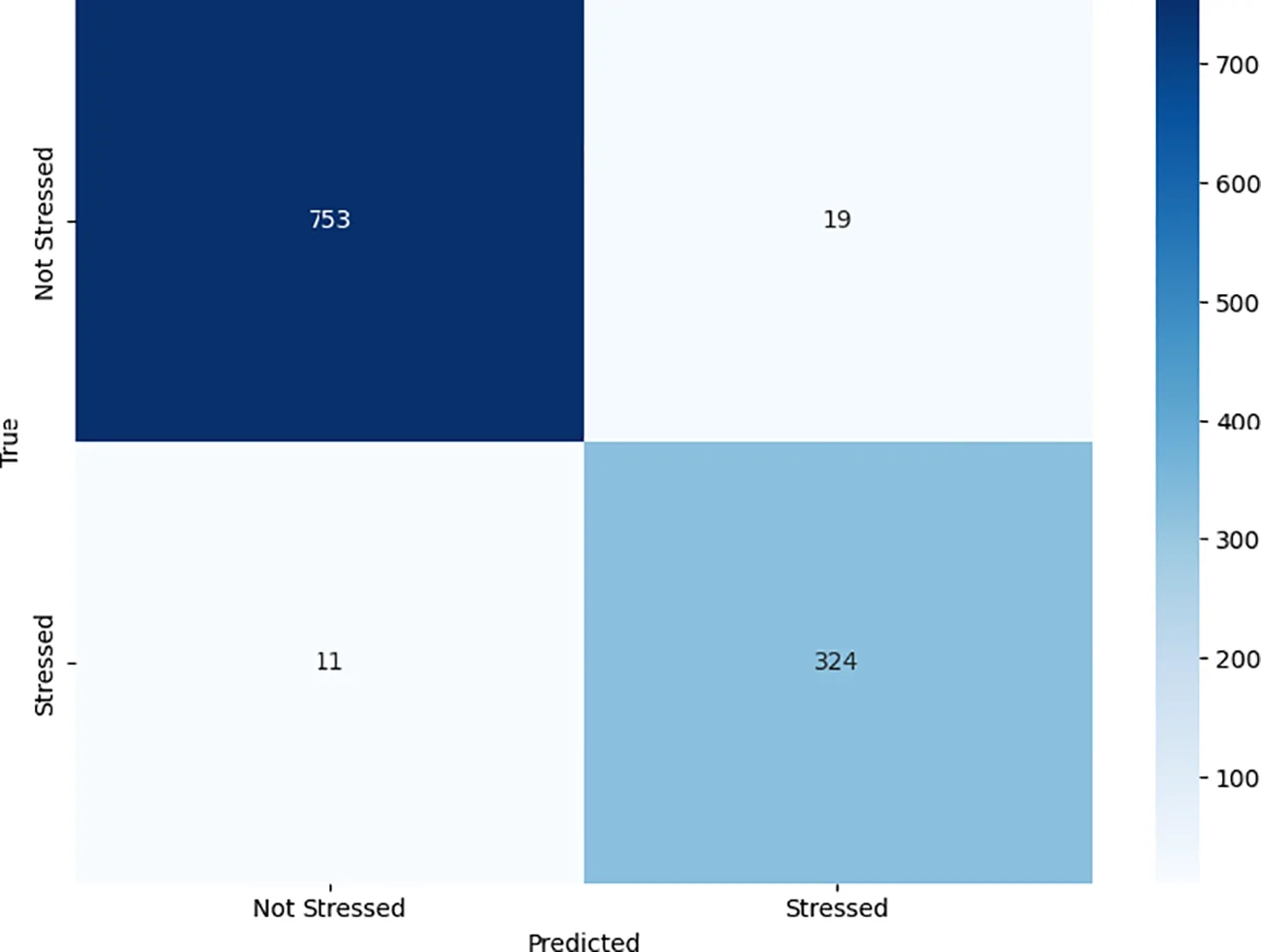
Confusion matrix of the proposed DT-GRU model on the held-out test set. Rows represent true labels, and columns represent predicted labels.

**Figure 4** presents the Receiver Operating Characteristic (ROC) curve of the proposed DT-GRU model on the held-out test set. The proposed DT-GRU model achieves an AUC of 0.9932, indicating nearly perfect discriminative power between stressed and non-stressed physiological states. The ROC curve shows a steep ascent in the upper-left corner of the graph (the ideal operating point). At a false positive rate of approximately 0.03, our model has a true positive rate of just above 0.99. The initial rise indicates that the proposed DT-GRU model detects most stressed windows while producing a few false alarms. According to the ROC curve for this framework, AUC = 0.9932, which is far from the diagonal dashed line (AUC = 0.50). It means the framework can discriminate between positive and negative samples across all classification thresholds.

**Figure 4:**
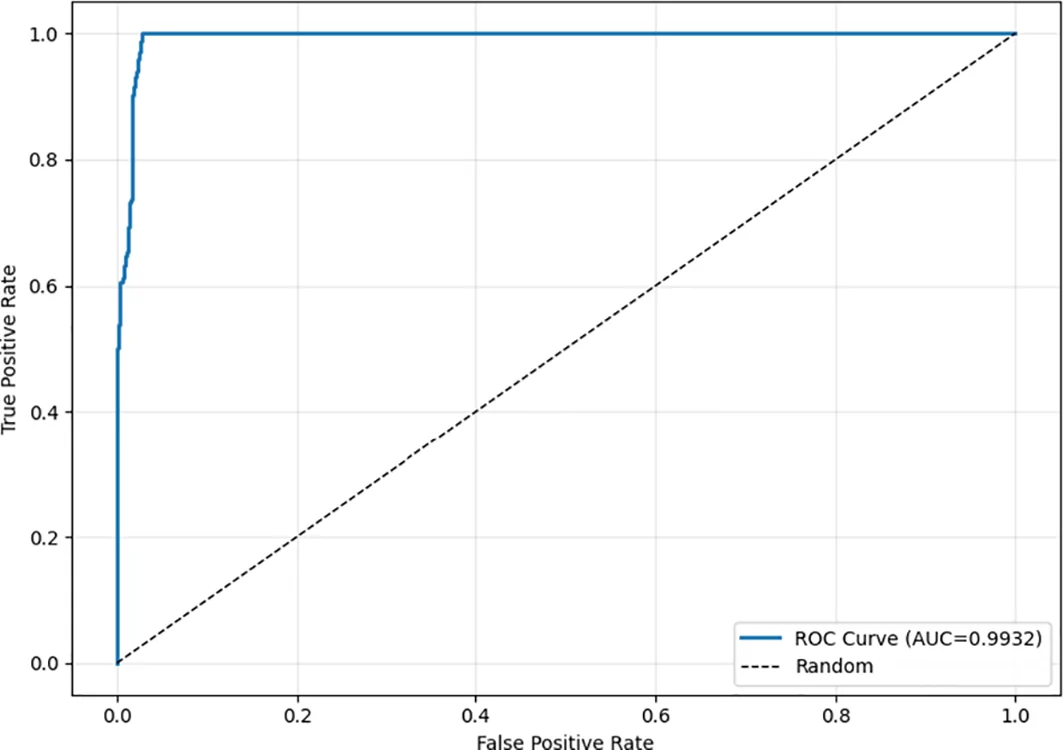
ROC curve of the proposed DT-GRU model on the held-out test set (AUC = 0.9932). The dashed diagonal line represents random classifier performance.

### 4.3 LOSO evaluation

**Table 3** presents the per-subject AUC scores obtained from the Leave-One-Subject-Out cross-validation experiment across all 15 WESAD subjects, and **Figure 5** illustrates the corresponding bar chart with the mean AUC line and standard deviation band.

**Table 3:** Per-subject AUC scores from LOSO cross-validation.

Subject	AUC
S2	1.0000
S3	0.6751
S4	1.0000
S5	0.9968
S6	0.9862
S7	0.9942
S8	0.9673
S9	0.9981
S10	1.0000
S11	0.7936
S13	1.0000
S14	0.6688
S15	1.0000
S16	0.9942
S17	0.1502
Mean AUC	0.8816
Std Dev	0.2268

**Figure 5:**
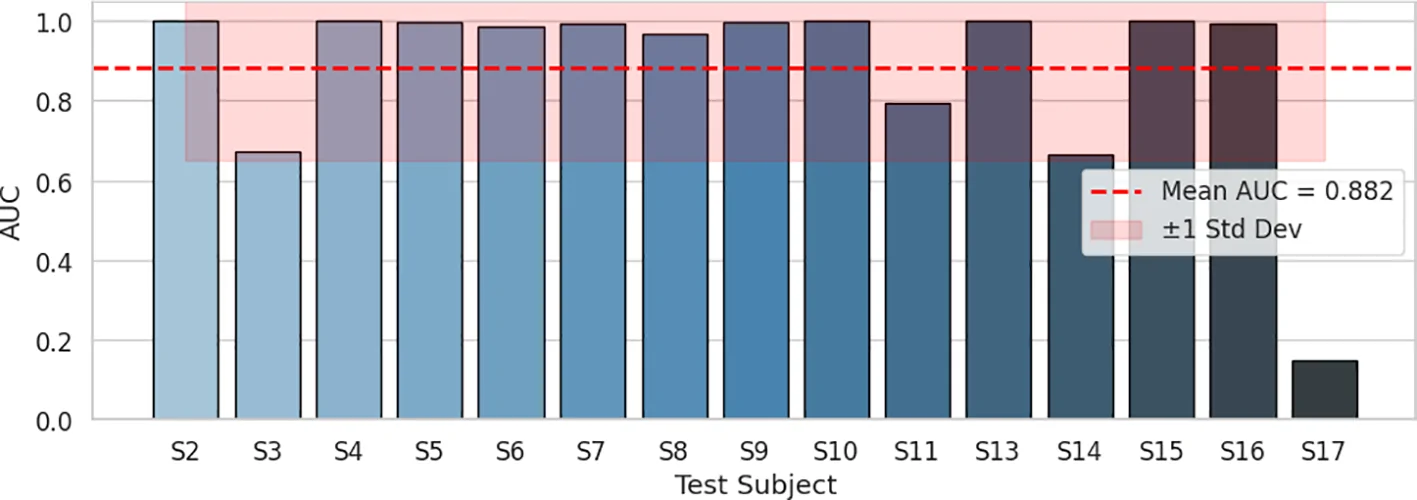
Leave-One-Subject-Out (LOSO) AUC performance across all 15 WESAD subjects. The red dashed line represents the mean AUC of 0.882, and the shaded pink region represents the ±1 standard deviation band.

As shown in **Table 3** and **Figure 5**, the proposed DT-GRU model achieves a mean LOSO AUC of 0.8816 ± 0.2268 in all fifteen subjects. Among the 15 subjects, 10 have an AUC greater than 0.96. As subjects S2, S4, S10, S13, and S15 have an AUC of 1.000, this indicates that DT embedding can capture and generalize physiological stress patterns for most subjects, including S5 and S6. The AUC scores for S7,

S8, S9, and S16 are also high (between 0.9673 and 0.9981), indicating good cross-subject generalization across most subjects. AUC scores of the three subjects are significantly lower: S3, 0.6751. S14, 0.6688. S17, 0.1502. S17’s poor performance with an AUC of 0.1502 is an outlier and the cause of this large std. dev., 0.2268. This variability at the subject level is consistent with the WESAD literature on inter-subject differences, in which certain subjects show physiological responses during the stress induction protocol that are atypical or non-discriminative and not shared by other subjects.

An ablation study was done to assess the embedding of the DT, the dimensionality of the embedding, and the depth of the GRU architecture on the same subject-independent evaluation protocol. As seen in **Table 4**, the results of the DT embedding ablation suggest that the performance boost given by the DT embedding is due to subject-level personalization and not just to the model capacity. A further variation of the embedding dimension reveals that an embedding dimension that is too small (8 dimensions) slightly reduces performance, while a larger embedding dimension (32 dimensions) yields a further performance drop, presumably because it becomes too large to represent the limited number of subjects in WESAD. The more complex the GRU model, the higher the accuracy of classification, but the number of parameters to be trained also increases significantly, from 25,073 to 92,337 parameters, when the GRU architecture is increased to GRU(128,64). Interestingly, the Single GRU(64) + DT-emb(16) configuration was found.

**Table 4:** Ablation study of DT-GRU architectural configurations under the subject-independent evaluation protocol.

Configuration	Parameters	Accuracy (%)	AUC
GRU(64,32) + DT-emb(16) [Proposed]	25,073	96.57	0.9941
GRU(64,32), No DT Embedding	24,321	96.30	0.9935
GRU(128,64) + DT-emb(16)	92,337	98.10	0.9971
GRU(64,32) + DT-emb(8)	24,697	96.48	0.9948
GRU(64,32) + DT-emb(32)	25,825	95.21	0.9928
Single GRU(64) + DT-emb(16)	16,689	97.29	0.9960

Minor variation from the 97.29% accuracy reported in Section 4.2 is attributable to a different random seed used in the ablation runs.

to be competitive in performance while using the fewest parameters, suggesting that DT embedding is also important in small architectures. In the end, the proposed GRU(64,32) + DT-emb(16) is a well-balanced configuration, offering good classification performance, computational efficiency, and suitability for deployment on wearable edge devices.

### 4.4 Discussion

The experimental results demonstrate that the proposed DT-Enhanced Gated Recurrent Unit (DT-GRU) framework achieves consistently high performance for binary stress detection on the WESAD dataset under both evaluation protocols. The standard subject-independent evaluation yields a test accuracy of 97.29% and an AUC of 0.9932, indicating that jointly modeling temporal physiological dynamics via the stacked GRU encoder and subject-specific personalization via the DT embedding yields a strong classifier. The model does so with only 25,073 trainable parameters, underscoring its computational efficiency and suitability for deployment in resource-constrained wearable sensing environments. The rapid convergence of the model, reaching peak validation performance at epoch 2 with a validation loss of 0.1059 before early stopping terminates training at epoch 7, suggests that the DT subject embeddings converge quickly and provide an effective personalized prior even with limited training data per subject. The consistently high validation AUC values throughout training, ranging from 0.9868 to 0.9973, further confirm that the model’s discriminative capability remains robust and does not degrade as training progresses, despite mild overfitting evident in the loss curve.

The confusion matrix results reinforce the clinical relevance of the proposed framework. With only 11 false negatives among 335 actual stressed windows, the model misses fewer than 3.3% of real stress episodes, which is a critical requirement for wearable health monitoring systems, where undetected stress events can have serious health consequences. The 19 false positives out of 772 non-stressed windows similarly represent a negligible false alarm rate of 2.5%, confirming that the model operates reliably across both classes despite the inherent 2.4:1 class imbalance in the dataset.

The LOSO cross-validation results reveal an important distinction between subject-dependent and subject-independent generalization. The mean LOSO AUC of 0.8816 ± 0.2268 reflects strong cross-subject generalization for the majority of participants, with 10 out of 15 subjects achieving an AUC above 0.96 and five subjects: S2, S4, S10, S13, and S15 attaining an AUC of 1.0000. However, the large standard deviation of 0.2268 is driven primarily by three underperforming subjects: S3 (AUC = 0.6751), S14 (AUC = 0.6688), and S17 (AUC = 0.1502). The particularly low AUC of S17 represents an outlier that substantially depresses the mean. This subject-level variability is consistent with well-documented inter-subject physiological heterogeneity in stress responses, where certain individuals exhibit non-discriminative or atypical autonomic patterns during the TSST stress induction protocol that cannot be reliably inferred from the physiological profiles of other subjects. These findings highlight the inherent challenge of cross-subject stress detection and suggest that future work should investigate adaptive personalization strategies such as few-shot calibration or subject-specific fine-tuning to improve performance on physiologically atypical individuals.

Because of the relatively small number of subjects in WESAD, the variety of physiological patterns available to train on may be limited, which can lead to a bias in training on subject-specific patterns, or to overfitting. Fortunately, there are data augmentation methods that can be applied to physiological time-series signals to address this and improve the model’s generalization. Specifically, adding Gaussian noise to the normalized training windows (where the window normalization is performed with a relatively low standard deviation, such as *σ* ≈ 0.01) can make the model more robust to sensor noise and inter-session variability, as is typical in real-world wearable deployments. Furthermore, the starting point of each training window may be jittered within a small window (e.g., ± 5 samples) to provide additional temporal diversity and reduce the system’s sensitivity to exact segmentation boundaries. The effectiveness of augmentation was assessed by conducting experiments on the augmented training datasets (Gaussian noise). The augmented DT-GRU model outperformed the original model, achieving a test accuracy of 97.65% and an AUC of 0.9967, whereas the original model achieved 97.29% accuracy and 0.9931 AUC. This is an absolute AUC gain of +0.0036, showing that low-complexity augmentation methods can improve the generalization performance. These findings indicate that this framework can be further strengthened to be robust in low-data regimes and motivate adding subject-aware augmentation strategies in next-generation extensions of this work, possibly in conjunction with the proposed framework.

Although the results of the experiments are very promising, the present study is limited in scope due to the small number of subjects in the WESAD dataset (only 15 subjects). WESAD is broadly accepted as a standard for wearable stress detection. All compared baseline methods are evaluated on the same data set, ensuring a fair comparison; however, the relatively small number of participants makes it difficult to draw general conclusions about the population. To overcome this limitation to some extent, a sliding-window segmentation approach was adopted, yielding 1,107 test windows and enabling the model to capture richer temporal stress patterns from the limited number of physiological recordings. However, future studies should test the proposed DT-GRU framework on more extensive and varied external datasets, including SWELL-KW and UBFC-Phys, to further evaluate its robustness, demographic generalization, and applicability to real-world deployment across varying recording scenarios and diverse populations.

Overall, the proposed DT-GRU framework demonstrates that incorporating a lightweight DT embedding into a GRU-based temporal encoder provides a principled and effective mechanism for subject-level personalization in wearable stress detection, achieving state-of-the-art classification performance on the WESAD benchmark while maintaining a compact, computationally efficient model architecture suitable for real-world deployment.

### 4.5 Feature importance analysis

The test set is used to determine the channel-wise permutation importances of the modalities most likely to influence the stress classification to make the proposed DT-GRU framework easier to understand, and to find the most valuable physiological modalities. The values of each physiological channel were independently set to zero for each test window, and the degradation in classification AUC was measured relative to the baseline model’s performance. The resulting feature importance is summarized in **Figure 6**.

**Figure 6:**
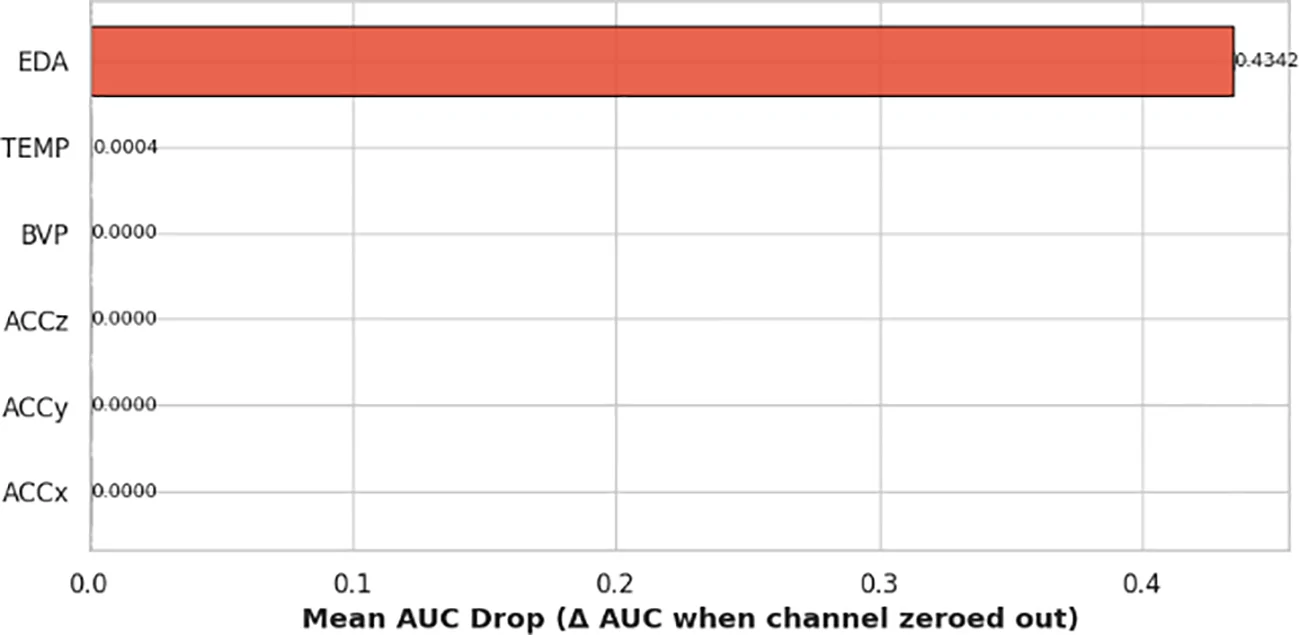
Channel-wise permutation feature importance analysis based on AUC degradation after independently perturbing each physiological modality. EDA exhibits the largest contribution to stress classification performance.

When removing a modality from the analysis, the analysis reveals that electrodermal activity (EDA) has the largest contribution to the stress classification performance, with an AUC reduction of 0.4342, significantly higher than all other modalities. This result is physiologically similar to previous studies, as EDA is a direct reflection of sympathetic nervous system activation and sweat gland activity, both of which are linked to psychological stress responses. There was a minimal but measurable contribution from the skin temperature (TEMP) channel, and negligible AUC degradation from the blood volume pulse (BVP) and accelerometer (ACCx, ACCy, ACCz) channels in the present experimental situation. The results indicate that the DT-GRU model primarily uses EDA signals to discriminate stress, while the other modalities provide supplementary information. Importantly, the feature importance analysis contributes to the clinical interpretability of the proposed framework by explicitly identifying the physiological signals that impact model predictions, which otherwise may be a concern as a deep learning-based wearable stress detection system may appear black-box.

### 4.6 Computational efficiency and edge deployment feasibility

A detailed computation efficiency analysis was performed to verify the suitability of the proposed DT-GRU framework for real-time wearable deployment. The model has 25,073 trainable parameters or around 97.94KB in float32 representation, indicating that the architecture is light enough to fit in the memory constraints of resource-constrained edge and embedded devices. The memory and computational footprints are much smaller than those of commonly used deep learning architectures for stress detection on wearable devices, such as CNN-LSTM and attention-based residual networks, which are much more complex. The inference latency was tested on the CPU with 1,000 test windows, and the average single-window inference time was 96.50 ± 4.46 ms, as shown in **Figure 7**. This results in a real-time processing system with less than 1% of the available decision budget per window (0.97%), allowing the model to perform well in real-time 557 constraints for continuous monitoring applications.

**Figure 7:**
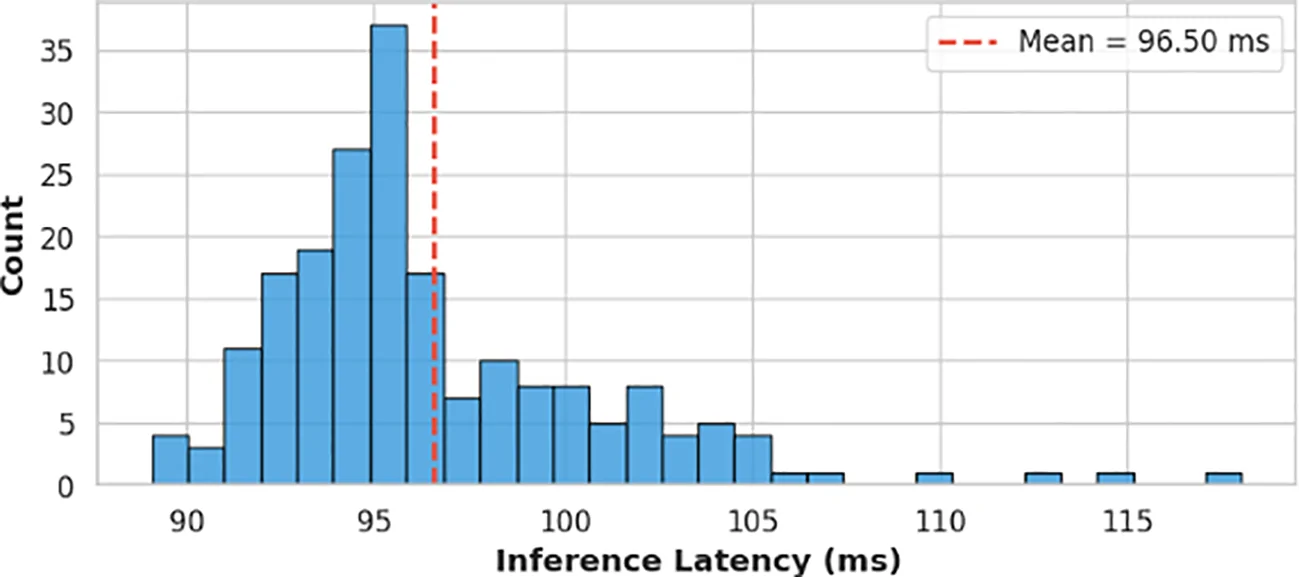
Distribution of single-window inference latency on CPU over 1,000 test samples, showing stable runtime performance with a mean latency of approximately 96 ms.

Moreover, the model deployment was successfully done to TensorFlow Lite (TFLite), resulting in the generation of a compact deployment artifact of 56.90KB without the use of custom operations, which makes it compatible with a standard mobile and edge inference pipeline. This allows deployment of the system on ARM-based devices, including Raspberry Pi, Android, and iOS devices, and potentially on energy-constrained wearable platforms. The inference throughput scales efficiently with batch size, with up to 500+ windows/second achievable in batch mode, further illustrating its suitability for real-time and near-real-time analytics. The proposed system provides competitive or higher stress classification accuracy with lower parameter complexity. The proposed DT-GRU framework is especially well-suited to practical implementation in continuous health monitoring systems where memory, latency, and energy are limiting factors.

## 5 Conclusion

This paper presented a Digital Twin-Enhanced GRU (DT-GRU) framework for personalized binary stress 569 detection on the WESAD dataset. By fusing a stacked GRU temporal encoder with a trainable subject embedding, the proposed model captures both physiological dynamics and inter-subject variability while remaining lightweight (25,073 trainable parameters). Across subject-independent evaluation, DT-GRU achieves 97.29% test accuracy and an AUC of 0.9932, and LOSO evaluation yields a mean AUC of 0.8816, highlighting strong generalization for most subjects. From a deployment perspective, the proposed DT-GRU architecture is well-suited for real-time wearable stress monitoring applications due to its low computational complexity and compact model size. After preprocessing, the physiological signals recorded in WESAD are sampled at 4 Hz, and the segmentation used is 30 seconds with a step size of 10 seconds. This means the system can compute a new stress prediction every 10 seconds, processing only the latest 30 seconds of physiological data, resulting in an accurate, low-latency, real-time inference pipeline for continuous monitoring applications. During a real-world deployment, physiological data collected from a wrist-worn wearable sensor can be sent over Bluetooth Low Energy (BLE) to a co-located smartphone application, and on-device inference can be performed using the DT-GRU model for each incoming sliding window. The GRU encoder would then continuously read the updated temporal buffer, and the DT embedding would provide lightweight personalization for the subjects during inference. Moreover, the compact design enables deployment on TensorFlow Lite (TFLite)- compatible devices, such as resource-constrained mobile and edge devices, with low memory and power consumption.

While WESAD is currently the community-approved benchmark dataset for wearable stress detection research, future studies will be conducted to validate the proposed DT-GRU framework on a larger and more ecologically representative dataset, to strengthen the robustness and real-world applicability. Specifically, cross-dataset evaluation will be performed on SWELL-KW (25 subjects, workplace stress conditions), UBFC-Phys (56 subjects, multimodal social stress, naturalistic interaction settings), and K-EmoCon (conversational, affective physiological recordings, naturalistic interaction settings).

## Data Availability

The original contributions presented in the study are included in the article/supplementary material. Further inquiries can be directed to the corresponding author.

## References

[B1] Bobade P. Vani M. (2020). “ Stress detection with machine learning and deep learning using multimodal physiological data”, in: 2020 Second International Conference on Inventive Research in Computing Applications (ICIRCA) (Piscataway, NJ: IEEE), 51–57.

[B2] Bolpagni M. Pardini S. Dianti M. Gabrielli S. (2024). Personalized stress detection using biosignals from wearables: A scoping review. Sensors 24, 3221. doi: 10.3390/s24103221 PMC11126007 38794074

[B3] Cellina M. Cè M. Alì M. Irmici G. Ibba S. Caloro E. . (2023). Digital twins: The new frontier for personalized medicine? Appl. Sci. 13, 7940. doi: 10.3390/app1313794030654563

[B4] Gil-Martin M. San-Segundo R. Mateos A. Ferreiros-Lopez J. (2022). Human stress detection with wearable sensors using convolutional neural networks. IEEE Aerosp. Electron. Syst. Mag. 37, 60–70. doi: 10.1109/MAES.2021.311519825079929

[B5] Katsoulakis E. Wang Q. Wu H. Shahriyari L. Fletcher R. Liu J. . (2024). Digital twins for health: a scoping review. NPJ Digital Med. 7, 77. doi: 10.1038/s41746-024-01073-0 38519626 PMC10960047

[B6] Lazarou E. Exarchos T. P. (2024). Predicting stress levels using physiological data: Real-time stress prediction models utilizing wearable devices. AIMS Neurosci. 11, 76. doi: 10.3934/neuroscience.2024006 38988886 PMC11230864

[B7] Li J. Washington P. (2024). A comparison of personalized and generalized approaches to emotion recognition using consumer wearable devices: Machine learning study. JMIR AI 3, e52171. doi: 10.2196/52171 38875573 PMC11127131

[B8] Malviya L. Mal S. Kumar R. Roy B. Gupta U. Pantola D. . (2023). “ Mental stress level detection using lstm for wesad dataset”, in: Proceedings of Data Analytics and Management: ICDAM 2022 (Amsterdam: Elsevier), 243–250.

[B9] Modi N. Kumar Y. Mehta K. Chaplot N. (2025). Physiological signal-based mental stress detection using hybrid deep learning models. Discover Artif. Intell. 5, 166. doi: 10.1007/s44163-025-00412-830311153

[B10] Mohd N. Sharma K. Salagrama S. Agrawal R. Patil H. (2023). Life span improvement of bio sensors using unsupervised machine learning for wireless body area sensor network. Rev. d’Intelligence Artificielle 37. doi: 10.18280/ria.370102

[B11] Moser M. K. Ehrhart M. Resch B. (2024). An explainable deep learning approach for stress detection in wearable sensor measurements. Sensors 24, 5085. doi: 10.20944/preprints202407.0707.v1 PMC11359526 39204782

[B12] Nandini D. Yadav J. Singh V. Mohan V. Agarwal S. (2025). An ensemble deep learning framework for emotion recognition through wearable devices multi-modal physiological signals. Sci. Rep. 15, 17263. doi: 10.1038/s41598-025-99858-0 40383809 PMC12086232

[B13] Nazeer M. Qayyum M. Patil G. Ali M. T. Srinivas J. Akheel M. (2024a). “ Stress detection based on ecg, gsr signals, hr, and behavioral context”, in: International Conference on Mathematical Modeling, Computational Intelligence Techniques and Renewable Energy (Cham: Springer), 351–366.

[B14] Nazeer M. Salagrama S. Kumar P. Sharma K. Parashar D. Qayyum M. . (2024b). Improved method for stress detection using bio-sensor technology and machine learning algorithms. MethodsX 12, 102581. doi: 10.1016/j.mex.2024.102581 38322136 PMC10844856

[B15] Schmidt P. Reiss A. Duerichen R. Marberger C. Van Laerhoven K. (2018). “ Introducing wesad, a multimodal dataset for wearable stress and affect detection”, in: Proceedings of the 20th ACM International Conference on Multimodal Interaction (New York, NY: Association for Computing Machinery (ACM)), 400–408.

[B16] Singh G. Chetia Phukan O. Gupta R. Nayyar A. (2024). Hybrid deep learning model for wearable sensor-based stress recognition for internet of medical things (iomt) system. Int. J. Commun. Syst. 37, e5657. doi: 10.1002/dac.565741531421

[B17] Sosa S. Fontecchio A. K. Chrysikou E. G. Atchison J. S. (2026). Beyond eda: A systematic review of multimodal sympathetic nervous system arousal classification for stress detection. Sensors 26, 1584. doi: 10.3390/s26051584 41829546 PMC12987280

[B18] Sun T. He X. Li Z. (2023). Digital twin in healthcare: Recent updates and challenges. Digit. Health 9. doi: 10.1177/20552076221149651 36636729 PMC9830576

[B19] Tanwar R. Phukan O. C. Singh G. Pal P. K. Tiwari S. (2024). Attention based hybrid deep learning model for wearable based stress recognition. Eng. Appl. Artif. Intell. 127, 107391. doi: 10.1016/j.engappai.2023.10739138826717

[B20] Tutunji R. Kogias N. Kapteijns B. Krentz M. Krause F. Vassena E. . (2023). Detecting prolonged stress in real life using wearable biosensors and ecological momentary assessments: naturalistic experimental study. J. Med. Internet Res. 25, e39995. doi: 10.2196/39995 37856180 PMC10623231

[B21] Vallée A. (2023). Digital twin for healthcare systems. Front. Digital Health 5, 1253050. doi: 10.3389/fdgth.2023.1253050 PMC10513171 37744683

[B22] Xiang J. Z. Wang Q. Y. Fang Z. B. Esquivel J. A. Su Z. X. (2025). A multi-modal deep learning approach for stress detection using physiological signals: integrating time and frequency domain features. Front. Physiol. 16, 1584299. doi: 10.3389/fphys.2025.1584299 40236827 PMC11997569

[B23] Yang S. Gao Y. Zhu Y. Zhang L. Xie Q. Lu X. . (2025). A deep learning approach to stress recognition through multimodal physiological signal image transformation. Sci. Rep. 15, 22258. doi: 10.1038/s41598-025-01228-3 40593851 PMC12216374

